# Constrained transcription factor spacing is prevalent and important for transcriptional control of mouse blood cells

**DOI:** 10.1093/nar/gku1254

**Published:** 2014-11-26

**Authors:** Felicia SL Ng, Judith Schütte, David Ruau, Evangelia Diamanti, Rebecca Hannah, Sarah J. Kinston, Berthold Göttgens

**Affiliations:** Department of Haematology, Wellcome Trust and MRC Cambridge Stem Cell Institute & Cambridge Institute for Medical Research, Cambridge University, Cambridge CB2 0XY, UK

## Abstract

Combinatorial transcription factor (TF) binding is essential for cell-type-specific gene regulation. However, much remains to be learned about the mechanisms of TF interactions, including to what extent constrained spacing and orientation of interacting TFs are critical for regulatory element activity. To examine the relative prevalence of the ‘enhanceosome’ versus the ‘TF collective’ model of combinatorial TF binding, a comprehensive analysis of TF binding site sequences in large scale datasets is necessary. We developed a motif-pair discovery pipeline to identify motif co-occurrences with preferential distance(s) between motifs in TF-bound regions. Utilizing a compendium of 289 mouse haematopoietic TF ChIP-seq datasets, we demonstrate that haematopoietic-related motif-pairs commonly occur with highly conserved constrained spacing and orientation between motifs. Furthermore, motif clustering revealed specific associations for both heterotypic and homotypic motif-pairs with particular haematopoietic cell types. We also showed that disrupting the spacing between motif-pairs significantly affects transcriptional activity in a well-known motif-pair—E-box and GATA, and in two previously unknown motif-pairs with constrained spacing—Ets and Homeobox as well as Ets and E-box. In this study, we provide evidence for widespread sequence-specific TF pair interaction with DNA that conforms to the ‘enhanceosome’ model, and furthermore identify associations between specific haematopoietic cell-types and motif-pairs.

## INTRODUCTION

Transcription factors (TFs) are primary mediators of gene regulation, and they have long been known as essential regulators of cell fate decisions in the haematopoietic system. TF proteins form complexes, bind regulatory DNA sequences on enhancers and promoter regions and help to recruit the basic transcriptional machinery to control the expression of nearby genes. The interaction between two TFs and the DNA therefore represents the most basic component in understanding larger TF complex formations (1). However, the molecular mechanisms by which such complexes control gene expression are still largely unknown. One of the best-understood enhancers controls expression of the interferon-β gene, where it is now recognised that specificity in gene expression does not arise from the cumulative effect of individual TF binding events but from synergistic effects of multiple TFs mediating the assembly of a higher order enhanceosome complex (2). Precise combinations of TFs as well as the orientation and spacing between TFs are therefore requirements for assembly of a transcriptionally active enhanceosome in this particular instance. However, a recent study in Drosophila suggests that none of the above requirements are prevalent in the majority of functionally validated enhancers. Instead, tightly controlled gene expression is postulated to be achievable using flexible spacing between TFs and redundancy in TF interaction (3).

Recent advances and improved cost-effectiveness in next generation sequencing technology have greatly increased the number of publicly available genome-wide TF binding profiles generated by chromatin immunoprecipitation coupled with sequencing (ChIP-seq). To date, hundreds of datasets exist in the public domain that have been generated by the haematopoiesis research community. We have previously described the development of the HAEMCODE compendium and web interface (4), which provides access for the wider scientific community to several hundred, carefully curated ChIP-seq datasets in mouse blood cells, thus enabling comparative analysis of datasets generated in multiple different laboratories. Similar resources have also been generated for embryonic stem cells (5,6).

In mouse haematopoiesis, specific spacing between two DNA binding motifs has previously been reported to be functionally important. Examples include (i) E-box and GATA motifs separated by 9bp and bound by TAL1 and GATA factors (7) that are important for the transcriptional activity of several erythroid gene regulatory elements and (ii) the Ets and IRF motif-pair separated by 2bp which occurs in gene regulatory sequences associated with genes important for lymphoid development (8). At genome-scale, similar analyses have been conducted on ENCODE datasets to show that binding of TF pairs can be spatially constrained (9,10). To assess the relative prevalence of spatially constrained binding versus the more relaxed model of the TF collective, genome-scale studies coupled with comprehensive statistical analysis and experimental validation will be required. Given the pivotal importance of combinatorial TF interactions in driving cell fate choices (11–13), research in this area not only has the potential to reveal new mechanistic aspects of TF function, but also inform our understanding of cell lineage specification during mammalian development.

Community efforts such as the development of HAEMCODE provide powerful new platforms for generating novel hypothesis that will lead to a better understanding of TF function. In this paper, we performed a comprehensive analysis of motif co-occurrence making use of all TF-datasets present in the HAEMCODE ChIP-seq compendium. By modelling TF binding to DNA with position weight matrices (PWMs), we were able to systematically predict binding sites and quantify the spacing between motifs. To infer TF pairs interacting with DNA, we chose an unbiased approach by considering not just sequence motifs that correspond to the TF precipitated in a particular ChIP-seq experiment, but also all other sequence motifs that were significantly enriched in the *de novo* motif discovery in a given sample. In this manner, we were able to comprehensively map instances of motif co-occurrence and quantify short range distances (±100 bp) between any pair of TFs across a large number of TFs and cell types. Statistical analysis indicated that TF partner choices are not random but are instead closely linked to cell-type-specific function. Moreover, experimental validation confirmed the functionality of two previously unknown motif-pairs and their spacing, involving the pairing of Ets with E-box and Ets with Homeobox TFs respectively.

## MATERIALS AND METHODS

### HAEMCODE ChIP-seq data processing

Public mouse ChIP-seq datasets from blood related cell types were obtained from the NCBI Gene Expression Omnibus and the EMBL-EBI European Nucleotide Archive (total: 289, Supplementary Table S1A). Raw reads were downloaded from the public repositories and then converted to *fastq* format and assessed for quality control using the FastQC software (http://www.bioinformatics.bbsrc.ac.uk/projects/fastqc). Adapter sequences were removed using the trimGalore software (http://www.bioinformatics.babraham.ac.uk/projects/trim_galore/). Reads from samples that pass the quality control were then aligned to the mm10 genome using Bowtie2 (14) and peak called using MACS2 (15) at different stringencies (*P*-value between 1e−4 and 1e−15). A suitable *P*-value was selected based on visual inspection of the ChIP-seq profiles in the UCSC genome browser. Supplementary Figure S1 shows the distribution of the number of peaks per sample for the 289 ChIP-seq samples.

### PWM similarity and clustering

Analysis of large numbers of DNA-binding motifs is difficult for several reasons. First, comparisons across hundreds of motifs would be impossible if some form of summarization is not employed. Second, motif databases are often redundant and since different TFs may bind the same motifs, they are often stored as separate motifs. To deal with the large scale and redundancy of motifs, we propose a method to cluster a collection of motif PWMs by their similarity. The web-based tool, STAMP (16) provide a solution to compare a set of query motifs against a motif database to obtain top PWMs that is most similar to each query motif. In contrast, to cluster a collection of motifs, an ‘all against all’ comparison is necessary. We developed a pipeline for performing PWM similarity clustering in R (http://www.r-project.org/) as follows. A distance score matrix, ***M***, was calculated for 240 Jaspar (17) PWMs where ***M*** is a matrix of size 240 × 240. The value for position (***a***,***b***) in the matrix is the mean Euclidean distance of the overlapping columns of a pair of PWMs, ***A*** and ***B***. See supplementary methods for details on computing the matrix, ***M***. Hierarchical clustering was applied to the score matrix and the resulting dendogram was written into a Newick tree file format. The Dynamic Hybrid algorithm in the *Dynamic Tree Cut* R package (18) was used to detect clusters in the hierarchical clustering results. Finally, the Newick tree file format and cluster memberships were uploaded to Evolview (http://www.evolgenius.info/evolview/) (19) to generate a circular cladogram. Source code for the PWM similarity clustering pipeline is available on bitbucket (https://bitbucket.org/feliciang/publication-motif-pair).

### *De novo* motif analysis and similarity to known motifs

The RepeatMasker program (http://www.repeatmasker.org) was used to assess the amount of repetitive elements in the 100bp sequences centred on the peak summit. Sequences containing >40% repeats were discarded and the remaining peak sequences were used for motif discovery. *De novo* motif analysis was carried out using the HOMER findMotifsGenome.pl program (20) and matches to known motifs were discovered using the TOMTOM software (21). Candidate *de novo* motifs were considered significant if they are present in at least 5% of the input sequences and have a p-value below the threshold of 1e−10. Matches to known motifs were discovered from the Jaspar v4 database (vertebrate CORE, FAM, PHYLOFACTS, POLII, CNE and SPLICE) (17) and only significant motifs (*Q*-value ≤ 0.05) were reported. In total, there were 1664 *de-novo* motifs and of these, 1152 had a significant match to Jaspar motifs.

### Motif-pair discovery

Our approach to discovering motif-pairs and significant motif spacing can be divided into two main tasks as denoted by (I) and (II) below. The first part (‘Scanning ChIP-seq peaks for known motifs’) describes the procedure to identify high-confidence binding sites of not just the TF precipitated in each experiment but also binding sites of regulatory partners. The second part (‘Finding motif-pairs and significant motif spacing’) outlines the procedure to find pairs of binding sites predicted in (I) that are up to 100bp apart and to discover significant spacing between high-confidence binding sites. We developed a custom analysis pipeline to address the above points and applied it to ChIP-seq peak regions in the HAEMCODE datasets. This approach allowed us to interrogate motif pairs with and without significant spacing in regulatory regions of mouse blood cells.

#### (I) Scanning ChIP-seq peaks for known motifs

To obtain the genomic coordinates of true, high-confidence binding sites, the HOMER findMotifsGenome.pl program (20) was executed with the ‘–find’ parameter on the 150bp region centred on the ChIP-seq peak summit. In contrast to the *de novo* motif analysis, we extended the search space by ±25 bp to take into account motifs that lie on the boundary of the 100 bp sequence. For each ChIP-seq sample, we scanned the peak sequences for Jaspar motifs identified as a significant match by TOMTOM in that sample so that only enriched motifs are taken into account. Collectively, all 289 samples encompass 354 enriched Jaspar motifs. We chose to use Jaspar motifs because PWMs of known motifs allowed comparisons to be made across datasets. The PWMs of *de novo* motifs, however, can appear in many ‘flavours’ (variations in PWM values for very similar sequence motifs) and presents a more difficult task for comparison.

When using the HOMER software to predict binding sites, two things needed to be considered for each PWM (i) ‘core’ motif and (ii) detection threshold. Firstly, the PWMs used to screen the input sequences are trimmed to obtain the ‘core’ motif. The ‘core’ motif is defined as the essential component of a motif that starts at the first position where information content (IC) ≥0.5 and ends at the last position where IC ≥0.5 (see Supplementary Methods for details on how to calculate IC). Therefore, PWMs are trimmed to obtain the motif ‘core’ by removing flanking regions with IC <0.5 and any motif ‘core’ <4 bp were discarded. The motif ‘core’ is then used for the screen rather than the full (original) motif to obtain a more accurate quantification of spacing between motifs. Without trimming, the flanking regions with low IC are treated as the full motif when screening and cannot be accounted for as true spacing (see Supplementary Figure S2). Second, a detection threshold value is calculated for each ‘core’ motif to predict high-confidence sites. See supplementary methods for details on calculating the detection threshold. For each ChIP-seq dataset, we scanned the 150bp peak sequences (centred on peak summit) using the ‘core’ motif and its detection threshold. Sequences that pass this threshold were considered high-confidence binding sites and used to find motif-pairs and motif spacing.

#### (II) Finding motif-pairs and significant motif spacing

First, the genomic locations for each Jaspar motif from the previous step (‘Scanning ChIP-seq peaks for known motifs’) with at least 50 occurrences in the genome were organized by Jaspar ID. By doing this, the motif regions are dissociated from the sample ID and unique coordinates were kept. By iterating over all pairwise combination of motifs, the offset, ***s***, between any two motifs was calculated using the BEDTools (22) function—windowBed—and then all motif-pair instances were grouped by its strand orientation (++, –, +–, –+) relative to the genome. For a pair of motifs—‘query’ and ‘target’, the offset value was obtained as follows. If the ‘query’ motif lies upstream of the ‘target’ motif, then the offset value is positive. On the other hand, if the ‘query’ motif lies downstream of the ‘target’ motif, then the offset value is negative. In both cases, offset is defined as the edge-to-edge distance between motifs and calculated starting from the first position flanking the ‘query’ motif to the first position (inclusive) of the ‘target’ motif (Supplementary Figure S2B). Without motif trimming, flanking regions may affect the offset value calculations.

The frequency, ***f***, of a particular motif-pair was then calculated for each offset value, ***s*** ∈ [–100, …, –1, +1, …, +100]. Significance tests were performed separately for each motif-pair, relative orientation (++, –, +–, –+) and offset value. Then, the significance of the observed frequency, ***f***, at a particular offset, ***s***, was tested separately under the assumption that each offset value is independent and has no effect on neighbouring values. Let ***N*** be the total number of observed motif-pairs with a specific orientation. The significance of ***f*** was tested using the binomial distribution if ***N*** ≤ 2000 or the Poisson distribution if ***N*** > 2000. Under the null hypothesis, a pair of motifs has no preferential spacing and, therefore, the expected probability of a motif-pair with a particular offset value (ranging from 1 to 100) in one of the four orientations is given by ${\boldsymbol q} = \frac{1}{{4*100}} = 0.0025$ and $\lambda = \frac{1}{{100}} \times N$, respectively. If there is no preference for motif spacing, the frequency of motif-pairs ***f*** with spacing ***s*** should follow a distribution

\begin{equation*} {\boldsymbol f}\sim {\rm Bin}({\boldsymbol N},\;{\boldsymbol q}),\;{\boldsymbol N} \le 2000 \end{equation*}

\begin{equation*} {\boldsymbol f}\sim {\rm Pois}(\lambda ),\;{\boldsymbol N} {>} 2000 \end{equation*}

Significant results were filtered using a *Q*-value (23) (http://www.bioconductor.org/packages/release/bioc/html/qvalue.html) threshold of 1e−4. Logos of motif-pairs were generated using the WebLogo software (version 3.3) (24). ‘Circos’ plots (25) were used to display unique motif-pairs (multiple offset value not considered) by motif cluster (described in the ‘PWM similarity and clustering’ section).

### Enrichment of motif-pair with significant spacing in ChIP-seq samples

To test the extent of overlap of motif-pair regions in HAEMCODE samples, the hypergeometric test was used to calculate the significance of the overlap. A ChIP-seq sample is significantly enriched for a motif-pair if the motif-pair genomic coordinates have a greater overlap with peak regions of a ChIP-seq sample than expected by chance. See supplementary methods for details on the hypergeometric test. Bonferroni correction (26) was applied to the p-value to correct for multiple motif-pairs and multiple ChIP-seq samples tested. For 7444 motif-pairs tested and 289 samples, we obtained a matrix of 7444 × 289 consisting of values 0 and 1: 1 denotes significance and 0 otherwise. A heatmap was generated in R using the *gplots* package (http://cran.r-project.org/package=gplots) by summarizing the matrix by cell type and motif-pair type. For each cell type category, we count the proportion of samples in a cell type that contain one or more motif-pairs in a specific motif-pair category (e.g. Ets + GATA) and coloured the heatmap cells based on this value. As a control cell type, we also performed the same enrichment analysis on 13 additional adipocyte ChIP-seq samples. Details of these 13 samples can be found in Supplementary Table S1B.

### SNPs and Indels

Data in vcf format was downloaded from the Wellcome Trust Sanger Mouse Genome Project version 3 (27). We used vcftools (28) to extract SNP and Indel features that overlap TF bound regions (ChIP-seq peaks). Box plots were generated in R using the *ggplot2* package (29).

### Candidate genes selection

For each candidate motif-pair, we searched for candidate genes for further testing as follows. Sequences of all motif-pair regions for a given candidate motif-pair were extracted in FASTA format and then aligned (without gaps) using the ClustalW2 software (30). New motif-pair PWMs were then calculated from the nucleotide frequencies of the aligned sequences. HOMER findMotifsGenome.pl was used to find the occurrences of motif-pairs based on the new motif-pair PWM. Finally, we mapped genomic regions of the motif-pairs to promoters of genes using annotation from MPromDb (31). For each candidate motif-pair, candidate genes were selected if they were expressed in any blood-related samples in the BioGPS dataset (32) and the conservation score (PhastCons or PhyloP) is within the top 10% amongst all mapped motif-pair occurrences. The BioGPS expression datasets was obtained from NCBI GEO (GSE10246). CEL files were processed by *gcrma* algorithm (33) in the gcrma R package. Conservation scores for 60-way Euarchontoglire multiple alignment were obtained from UCSC (http://hgdownload.cse.ucsc.edu/goldenPath/mm10/).

### Transient luciferase assays

Wild-type and mutant DNA fragments for candidate regulatory regions (*Atf3*, *Cbfa2t3* and *Csf3* promoters) were obtained from GeneArt^®^ by Life Technologies (see Supplementary Figure S3) and cloned into the pGL2 basic vector from Promega. K562 and 416b cell lines were transfected with the relevant vectors and a lacZ control vector by electroporation (220 V, 900 μF). Experiments were performed in triplicate and each experiment contained three technical replicates. The luciferase activity was analysed 24 h after transfection using the FLUOstar OPTIMA luminometer from BMG LABTECH. Significance was calculated by combining the *P*-values of each experiment (generated by using the *t*-test function in Excel) using the Fisher's method.

## RESULTS

### Public ChIP-seq datasets represent a rich resource for genome-wide motif-pair discovery

**Figure 1.**
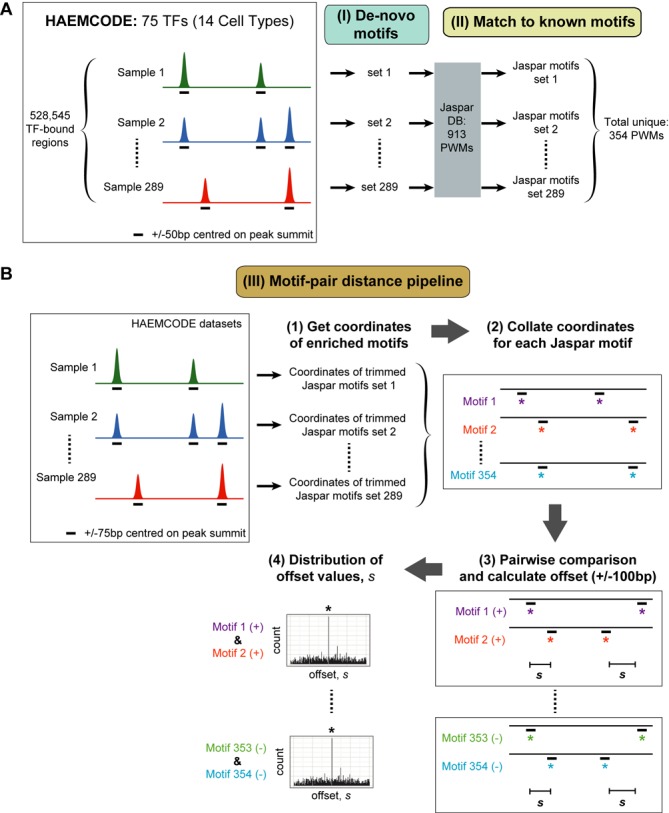
Workflow of this study. (**A**) Motif analysis procedure applied to 289 HAEMCODE datasets. *De novo* motif analysis was carried out separately for each sample in HAEMCODE. Each individual set of significant *de novo* motifs was then independently compared to the Jaspar collection of known motifs (total: 913 PWMs). Collectively, 354 unique PWMs were identified as significant matches within the 289 datasets. These unique PWMs were used in the motif-pair distance pipeline. (**B**) Procedure for identifying motif-pairs and over-represented distances between motifs. TF-bound regions (±150 bp centred on the peak summit) for each sample were individually scanned for the corresponding set of ‘trimmed’ Jaspar motifs so that only enriched motifs in that sample were considered (step 1). Then the coordinates of enriched motifs for all 289 samples were compiled and organized by motif ID (step 2). At this stage, the motif coordinates are dissociated from the sample ID. For all possible pairwise combinations of motifs and orientation, the offset values between motifs, ***s***, were calculated and significant offset values within ±100 bp were identified (steps 3 and 4).

We previously reported the development of the HAEMCODE repository for curated public ChIP-seq datasets in mouse blood cells (4). This large collection of TF-binding maps is a rich resource for genome-wide analysis of gene regulation in haematopoiesis. Several hundred TF ChIP-seq samples covering all major blood cell types have been made publicly available on HAEMCODE for the scientific community. All datasets included in the HAEMCODE repository have been processed using a standardized ChIP-seq analysis pipeline (see ‘Materials and Methods' section). At the start of this study, 289 ChIP-seq datasets (Supplementary Table S1A) covering 75 TFs across 14 blood cell types had been processed and so we focussed our analysis on this set of samples which in total corresponded to 528 545 genomic regions bound by at least one TF. For each ChIP-seq sample, we performed *de novo* motif analysis using HOMER (20) on the 100 bp sequences centred on the peak summit (Figure 1A). Enriched DNA motifs for each sample were then analysed using the TOMTOM program (21) to identify significant matches to known motifs in the Jaspar library (total of 913 PWMs) (17). Collectively, the enriched motifs in all 289 samples had significant matches to 354 Jaspar PWMs. These motif discovery results are the basis for the integrative analysis presented below.

The spacing between TF binding sites can be an important feature of TF co-occupancy. We reasoned that combining results from 289 ChIP-seq studies should allow us to perform a deep analysis of this phenomenon. Several studies in recent years have reported examples of preferred distance requirements between pairs of TFs binding on DNA using ENCODE datasets (9,10). Moreover, E-box and GATA binding sites separated by nine nucleotides have long been recognized as an important regulatory feature in erythroid and megakaryocytic cells (7). To examine TF co-occupancy and motif spacing preferences in the entire genome of haematopoietic cells, we developed a motif-pair discovery pipeline to identify motif co-occurrences and preferential distance(s) in all 528 545 TF-bound regions (Figure 1B). We used ‘trimmed’ Jaspar motifs (see methods) that are enriched in a particular sample to scan the TF-bound regions (150 bp centred on peak summits) of that sample and obtain genomic coordinates of motif occurrences. Of note, this procedure was performed independently for each sample using only those Jaspar motifs that were over-represented in the given sample in order to minimize the number of false positive results and to allow comparison across multiple datasets.

In total, 354 Jaspar motifs were used in the scan, so genomic coordinates from the scan were collated and organized by the 354 Jaspar IDs tested. Next, a comprehensive search was carried out for all combinations of motif-pairs and the relative orientations of the motif. For each motif-pair and orientation, the offset values between these two motifs (within ±100 bp) were calculated. Finally, all offset values from −100 to −1 and +1 to +100 were examined to identify distances that are over-represented and favoured by the motif-pair (see ‘Materials and Methods’ section). Taking into account distinct motif-pairs, orientation and preferential offset values, 7444 significant results were obtained at a *Q*-value threshold of 1e−4 and these results (motif-pair logos and genomic coordinates) have been made available on this website—http://bioinformatics.stemcells.cam.ac.uk/publications/motifpair/motifpair.html. Processed ChIP-seq data, *de novo* motif and significant matches to Jaspar motifs for the 289 samples are also available and can be accessed from the HAEMCODE website—http://codex.stemcells.cam.ac.uk/.

### Preferred spacing of TF binding site pairs is prevalent in haematopoietic TF-bound regions

**Figure 2.**
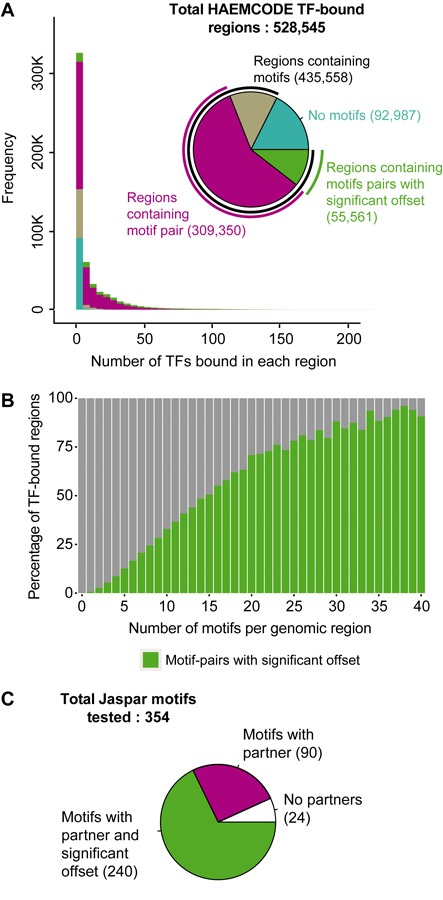
Motif-pairs with preferential spacing are prevalent in haematopoietic TF-bound regions. (**A**) The pie chart shows all genomic regions in this study bound by at least one TF and the proportion of regions containing motifs or motif-pairs (with and without significant spacing). The histogram shows the distribution of the number of TFs (binwidth = 5) in each genomic region and each bar is coloured to indicate the presence or absence of motifs and motif-pairs (with and without significant spacing). (**B**) Barchart indicates the percentage of TF-bound regions (*y*-axis) that contain motif-pairs with significant offset. Percentages are shown for TF-bound regions containing zero up to 40 motifs (*x*-axis). (**C**) All unique Jaspar motifs identified as significant matches to *de novo* motifs in Figure 1A (step II). These motifs were used to scan TF-bound regions for binding sites and the pie chart highlights the proportion that participates in motif-pairs (with and without significant spacing).

By integrating Jaspar motif analysis results from the 289 HAEMCODE TF-binding maps, we were able to study the TF co-occupancy patterns and preferential spacing that are relevant to haematopoiesis. In total, we analysed 528 545 regions that are bound by at least one TF in any of the 289 datasets (overlapping regions were merged). Out of all these regions, 435 558 (82.4%) regions contain an over-represented motif and 364 911 (69%) regions contain motif-pairs (Figure 2A). This suggests that a majority of experimentally verified TF binding regions are most likely also bound by another TF. Of the 364 911 regions containing motif-pairs, 55 561 regions (15.2%) contain motif-pairs that have a recurring, significantly over-represented preferential spacing between the two motifs. Although a large proportion of the TF-bound regions were only bound by one TF (218 332, 41.3%), motif-pairs with significant spacing were also discovered in these regions (4170, 1.9%). This result indicates that experimental data for certain TFs are still missing and, therefore, not yet included in the compendium. Interestingly, we also found that 17.6% (92 987 regions) of all TF-bound regions contain no match to any of the 354 motifs analysed. The vast majority (92.5%, 86 056) of these ‘motif-less’ TF-bound regions was bound by just a single TF.

**Figure 3.**
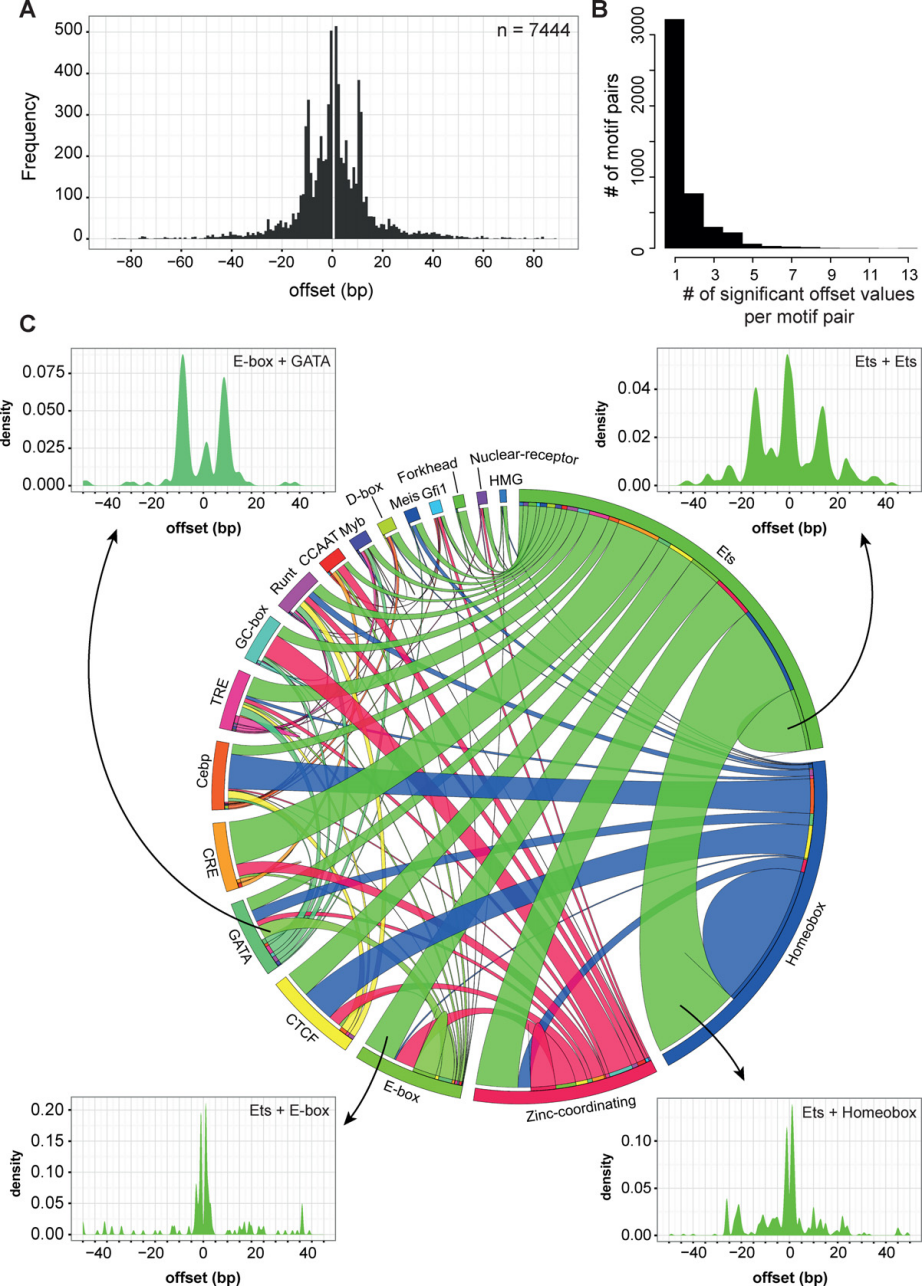
Motif-pairs with significant spacing. (**A**) Distribution of all significant offset values for all motif-pairs and motif orientations. (**B**) Number of significant offset values per motif-pair. The number of offset value refers to the total unique offset values for a specific motif-pair of a specific orientation. (**C**) ‘Circos’ plot showing all unique motif-pairs and orientations with significant offset value grouped by motif cluster. Clusters were defined by similarity and clustering of Jaspar PWMs (see Supplementary Figure S5) and arranged on the plot based on the number of pairs per cluster. Ribbon thickness is proportional to the number of unique motif-pairs (multiple offset values not counted) belonging to the clusters. Data used to generate ‘circos’ plot can be found in Supplementary Table S2. Example offset density plots in the range of ±50 bp are shown for four types of motif-pairs to illustrate distinct preferences for spacing between motifs: ‘E-box + GATA’ (28 unique pairs), ‘Ets + E-box’ (67 unique pairs), ‘Ets + Ets’ (126 unique pairs), and ‘Ets + Homebox’ (182 unique pair).

TF-bound regions with more motifs are more likely to contain motif-pairs with significant offset (Figure 2B) suggesting that preferential spacing is frequently found in regions where many TFs co-operate. A large proportion of regions that contain motif-pairs with preferential spacing are located within intragenic regions (24 751, 38.1% ), but a large proportion is also found in intergenic regions (23 578, 36.3%). Motif-pairs are also common in promoter regions (10 755, 16.6%), especially considering that only 5.4% of all TF peak regions overlap with promoters (Supplementary Figure S4A and B). A small proportion of motif-pair regions are found within UTRs and exons (Supplementary Figure S4B). When compared to data from the Vista Enhancer Browser (34), 13.6% (172/1261) of known enhancers in this database overlapped motif-pair regions with constrained spacing from our dataset. Presumably the overlap is not larger because the Vista database contains a relatively small set of validated enhancers mainly related to brain, muscle and limb development. In total, 354 Jaspar motifs were enriched in at least one sample and of these, 330 motifs (93.2%) were found to participate in a pair (Figure 2C). Of these 330 motifs, 240 motifs (72.7%) contributed to motif-pairs with significant preferential spacing and this subset covers all TF classes in the Jaspar library (Supplementary Figure S4C). Overall, the distribution of all significant offset values shows a symmetric distribution with preferential offset values of ∼±1 and ∼±11 bp (Figure 3A); with the former consistent with directly adjacent binding of two TFs, whereas the latter corresponds to one helical turn around the DNA. Furthermore, motif-pairs appear to require a strict spacing with the majority of pairs having just one preferred offset value (3216, 69.1%) (Figure 3B).

To facilitate interpretation of this large number of results, we next clustered the Jaspar PWMs into groups of similar motifs. Jaspar's collection of motifs is highly redundant and often more than one PWM exists for the same type of binding site. The 240 motifs that participate in a motif-pair with preferential spacing were processed through a newly developed clustering procedure (see methods for algorithm and link to R source code) to obtain 19 clusters of similar PWMs (Supplementary Figure S5). Thirty four PWMs did not fall into any of the 19 clusters of major motif classes and were removed from further analysis, but are available in Supplementary Table S3. By grouping all significant motif-pairs by the cluster they belong to, we were able to display all the interactions between motif clusters in a ‘circos’ plot (Figure 3C). The ribbon thickness in the plot is proportional to the number of unique motif-pairs (multiple offset value not counted) found between the two clusters of motifs and some of the most abundant types of motif-pairs are shown by thick ribbons. This also reflects the abundance of certain types of motif-pairs in haematopoietic transcription. Examples of heterotypic motif-pairs (two different motifs) include ‘Ets + Homeobox’ (182 unique pairs), ‘Cebp + Homeobox’ (71 unique pairs), ‘CTCF + Homeobox’ (67 unique pairs) and ‘Ets + zinc-coordinating’ (86 unique pairs). Partnering between motifs from the same cluster (e.g. homotypic motif-pairs), is also possible and this is shown by semi-circles within the same cluster of motif. For example, the ‘Ets + Ets’ type of motif-pairs constitute ∼15.1% of all the motif-pairs involving an Ets motif, while ‘Homeobox + Homeobox’ make up 42% of all Homeobox motif-pairs.

It is also worth noting that four motif clusters—Ets, Homeobox, Zinc-coordinating and E-box, have high numbers of interacting partners and make up >50% of all motif-pairs. For example, Ets motifs can form pairs with all the other clusters of motifs and with itself while, in contrast, CRE motifs form pairs almost exclusively with Ets motifs. When examining specific motif-pairs, we found distinct preferences in the significant offset values. As shown in the four example density plots (Figure 3C), the ‘E-box + GATA’ pair are separated by ∼9 bp, ‘Ets + E-box’ pairs are separated by ∼1 bp, ‘Ets + Ets’ are separated by ∼1 or ∼15 bp, and ‘Ets + Homeobox’ are separated by 1–3 bp. Importantly, this analysis also demonstrated the consistency of our results despite utilizing different ‘flavours’ of the same motifs in the Jaspar library because the same offset values are frequently significant across independent motifs within each cluster pair. Since we analysed the motifs in relation to genome orientation, our results are symmetric in that offset values that were significant in the ‘plus’ strand were also significant in the ‘minus’ strand. The full results for all heterotypic and homotypic motif-pairs and their preferential spacing can be found in Supplementary Figure S6.

### TF-bound regions containing motif-pairs are functionally important

**Figure 4.**
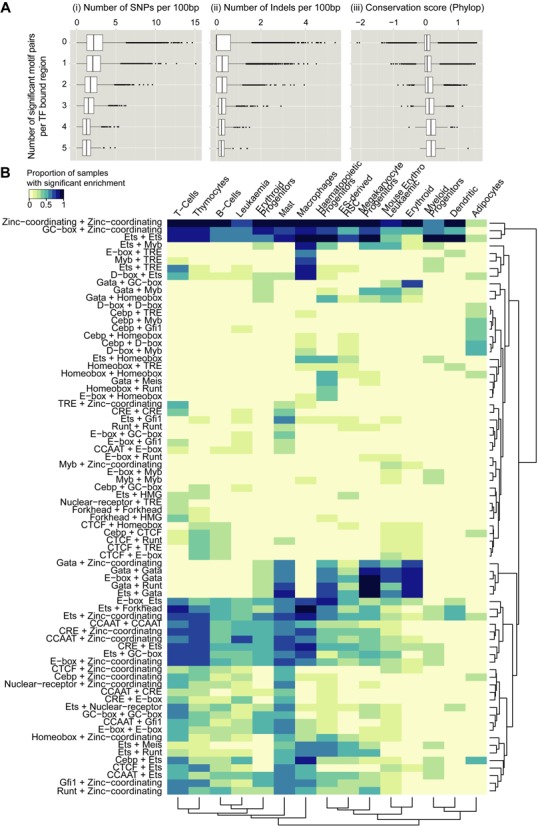
Properties of motif-pairs and orientation with significant spacing. (**A**) (i) Box plot showing the distribution of the number of SNPs overlapping TF-bound regions (ChIP-seq peaks) containing zero to five motif-pairs. (ii) Same as in (i) but for Indels. (iii) Same as in (i) but for PhyloP conservation scores. Positive PhyloP scores indicate conservation in 60way Euarchontoglire multiple alignment. (**B**) Heatmap of significantly enriched motif-pairs by cell type. Pairs of motifs were grouped by motif clusters they belong to (see Supplementary Figure S5). For a particular pair of motif cluster, row elements are coloured based on the proportion of samples in each cell type that are enriched for at least one motif-pair in that category. Only motif-pair categories that are significantly enriched in ≥20% samples in at least one cell type are shown. Dark blue cells denote higher proportion; light yellow cells denote lower proportion.

Having shown that the spacing between motif-pairs is commonly constrained, we next made use of the organization of results by motif clusters to determine patterns that are consistent and therefore most likely to be biologically relevant. To investigate further the functional role of motif-pairs and their preferred spacing, we examined single nucleotide polymorphism (SNP) and insertion/deletion (Indel) data from the genomes of 18 key mouse strains (27). We reasoned that if motif-pairs with preferential spacing are functionally important, these regions are under stronger selective pressure compared to regions without motif-pairs. Indeed, we found that regions containing one or more overlapping motif-pairs have fewer SNPs and Indels than regions without motif-pairs (Figure 4A (i) and (ii)). Moreover, there are fewer SNPs/Indels found in regions in which more motif-pairs are present. The conservation score on these regions also shows that the number of motif-pairs is directly proportional to higher conservation score (Figure 4A (iii)). Of note, this trend was not observed when we examined TF-bound regions containing zero up to ten motifs regardless of the number of motif-pairs with constrained spacing (Supplementary Figure S8A). To test the significance of the observed trend, we used the Kolmogorov–Smirnov test to compare the distribution of values (SNP, Indel, PhyloP) in TF-bound regions containing 0 motif pairs against TF-bound regions containing 1 or more motif-pairs. Results showed that the two distributions are indeed very different (Supplementary Figure S8B) and, therefore, represent two different types of regulatory regions where regions with more motif-pairs are more conserved. These results suggest that regulatory regions that contain spatially constrained motif-pairs are less likely to be mutated, and therefore are likely to be particularly important for haematopoietic gene regulation.

We also investigated the role of motif-pairs with preferential spacing in relation to cell-type-specific functions in blood development by analysing the enrichment of motif-pairs in peak regions of all 289 samples. For each motif-pair with significant offset value, we tested each of the 289 samples independently for over-representation of the motif-pair in the sample and summarized the results in a heatmap (Figure 4B). The 289 samples were categorized by cell types (heatmap columns) and motif-pairs were categorized by motif clusters (heatmap rows) to illustrate the proportion of samples in a cell-type enriched with a motif-pair. The heatmap revealed several interesting patterns about the relationships between motif-pairs and stages in blood development. Three motif-pairs—‘zinc coordinating + zinc coordinating’, ‘GC-box + zinc coordinating’, and ‘Ets + Ets’ (heatmap rows 1–3)—are significantly enriched across all haematopoietic cell types. In contrast, five pairs involving GATA motifs—‘GATA + zinc coordinating’, ‘GATA + GATA’, ‘E-box + GATA’, ‘GATA + Runt’ and ‘Ets + GATA’ (heatmap rows 46–50)—are more cell-type-specific, as shown by their enrichment in haematopoietic progenitors as well as cells of the erythroid and myeloid lineage. Moreover, when we performed the same analysis on adipocyte ChIP-seq samples, blood-related motif-pairs have very low enrichment in this cell type. To further examine the cell type-specific properties of motif-pairs, we explored an alternative visualization of the motif-pairs enriched in different cell types by using ‘circos’ plots to display the abundance of motif-pairs in each cell type (Supplementary Figure S7). We find that ‘Homeobox + Homeobox’ motif-pairs are predominantly found in multipotent haematopoietic progenitors and ES-derived HSPCs. In all cell types, CTCF is an important TF binding partner as shown by the ribbons extending from CTCF to several other motifs. We also observed that motif-pairs involving ‘GATA’ are mostly found in haematopoietic progenitors and cells of the erythroid and myeloid lineage.

### Exact spacing between motifs is important for transcriptional activity

**Figure 5.**
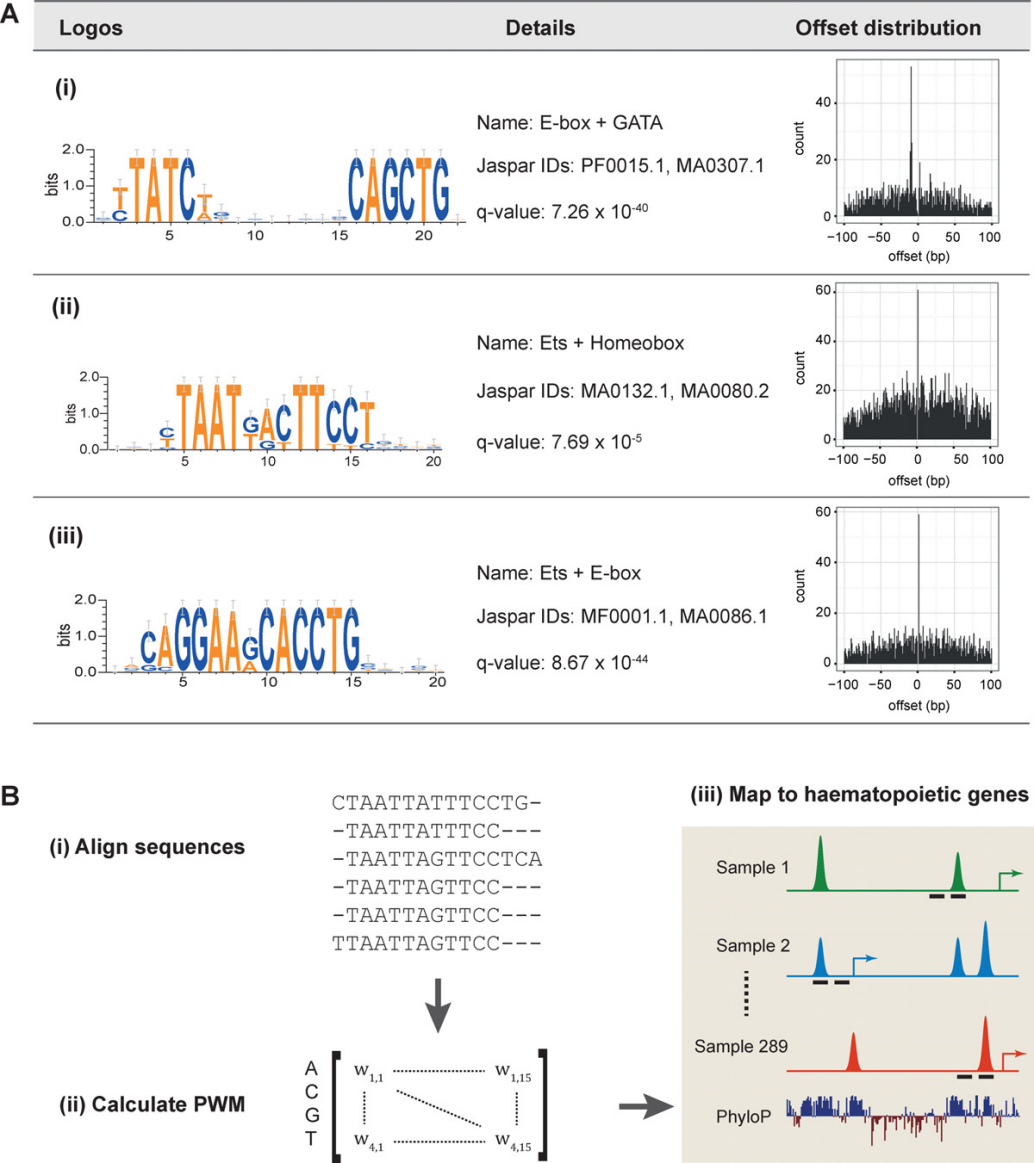
Candidate motif-pairs. (**A**) Logos, details and offset distribution of three candidate motifs. Each candidate was named based on the motif clusters it belongs to. (**B**) Procedure for identifying candidate genes for functional analysis. For each candidate motif-pair, a PWM was calculated based on the alignment of all the corresponding motif-pair regions. The new motif-pair PWMs were then used to map to conserved haematopoietic genes (see ‘Materials and Methods' section).

To investigate the role of strict spacing between TFs, we selected three candidate motif-pairs: ‘E-box + GATA’, ‘Ets + E-box’, and ‘Ets + Homeobox’ (Figure 5A). We selected these motif-pairs because ‘E-box + GATA’ is a well-known motif-pair in haematopoiesis, while the latter two are previously unknown motif-pair spacings. Moreover, these three candidates are among the most frequently occurring results. As shown by the density plots in Figure 3C, these motif-pairs favour ∼9, ∼1 and ∼3bp offset values, respectively. GREAT analysis on these motif-pair regions showed enrichment of blood-related processes, phenotypes and diseases (see Supplementary Table S4).

**Figure 6.**
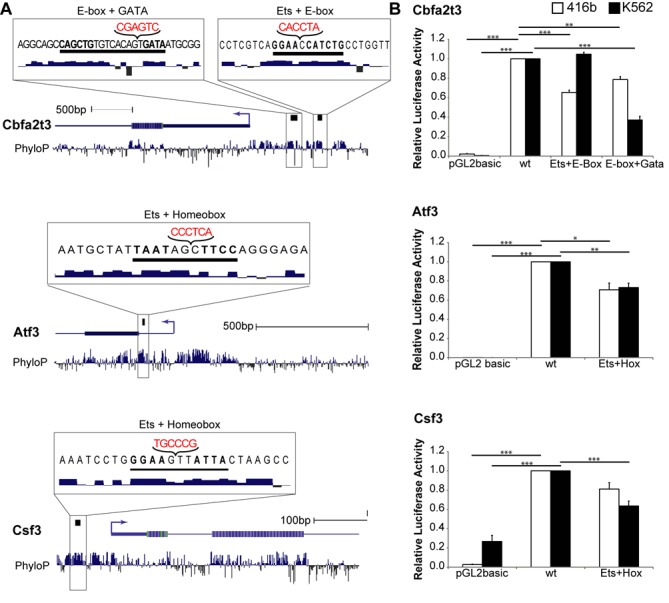
Functional validation of candidate genes. (**A**) UCSC genome browser tracks showing promoter regions of Cbfa2t3, Atf3 and Csf3 genes. The candidate motif-pairs for each gene are highlighted on top of each gene and enlarged in the genome browser track inset. The nucleotides that were introduced in order to disrupt the spacing between the two motifs are shown in red. (**B**) Luciferase assays in transiently transfected 416b or K562 cells. Results indicate the average and standard error of the mean of three independent repeats and are shown relative to the luciferase activity of the wild-type promoter (*P*-values: * ≤0.05, ** ≤0.01, *** ≤0.001).

We chose TF-bound regions for functional validation by performing a screen on all HAEMCODE regulatory regions for occurrences of the candidate motif-pairs using the newly generated PWMs for each candidate motif-pair (Figure 5B). We calculated each motif-pair PWM from the aligned sequences of motif-pair regions and the new probability matrices can be found in Supplementary Table S5. Candidate genomic regions were prioritised based on two criteria: (i) high evolutionary sequence conservation and (ii) expression of the adjacent gene in one or more blood-related cell types. Following the screen, we chose the promoter regions of the following three genes for further analysis: *Cbfa2t3*, *Atf3* and *Csf3* (Figure 6A). The *Cbfa2t3* transcriptional co-repressor interacts with TAL1 to regulate erythrocyte differentiation (35,36) while *Atf3* is a key transcriptional repressor of target genes in lipopolysaccharide-stimulated macrophages (37). Macrophage progenitor function has also been shown to be dependent on *Csf3* because mice with *Csf3* deficiency produced lower levels of granulocyte and macrophages compared to mice without the deficiency (38). To investigate the importance of motif spacing, we generated luciferase reporter constructs containing wild-type and motif-spacing mutant versions of these three promoters. Motif-pair spacing was disrupted by introducing a 6bp random sequence (see ‘Materials and Methods' section) and the random sequences were designed without generating additional motifs while maintaining the flanking nucleotides, and in all cases maintained at least one nucleotide flanking the motifs. Retaining one nucleotide may not be sufficient in situations where the offset is more than one base pair, suggesting that the design of the random sequence may be improved in future analysis by retaining two or more nucleotides. Luciferase reporter gene assays demonstrated that the 9 bp spacing between the GATA and E-box motifs within the *Cbfa2t3* promoter region is important for transcriptional activation in human (K562) as well as murine (416 bp) cell lines because disruption of the 9 bp spacing caused a significant reduction in the luciferase activity in both cell lines (Figure 6B). These results established the *Cbfa2t3* promoter region as a new example of haematopoietic regulatory elements that depend on the 9bp-spaced ‘E-box + GATA’ motif-pair. Importantly, similar analysis of the newly identified motif-pairs between Ets and Homeobox as well as Ets and E-box motifs revealed important functions for gene activation of *Atf3*, *Csf3* and *Cbfa2t3* in mouse and/or human cells. Indeed, luciferase activity of motif-spacing mutants was significantly reduced for all tested promoter regions in at least one of the two cell lines (Figure 6B). In the *Atf3* promoter region, luciferase activity for the Ets and E-box pair did not change in K562 cells although a significant reduction was observed in 416 bp cells, therefore suggesting that Ets and E-box TFs may not form a complex on the *Atf3* promoter in K562 cells. Luciferase assays therefore provided functional validation of two previously unrecognized constrained motif-pairs, namely the ‘Ets + Homeobox’ and ‘Ets + E-box’ motif-pairs. Going back to the HAEMCODE compendium, we also found evidence for binding of E-box, GATA and Ets TFs on the motif-pair regions on the *Cbfa2t3* gene locus (Supplementary Figure S9).

## DISCUSSION

The widespread uptake of ChIP-seq technology has undoubtedly improved our understanding of multiple aspects of TF function in mouse haematopoiesis, such as combinatorial aspects of transcriptional control in HSCs (39), function of key regulators such as Gata3 across the T-lymphoid lineage (40), early acquisition of blood progenitor transcriptional programs (41,42) and the identification of different TF binding modes for a given factor such as TAL1 (43). Focussed studies by individual laboratories are complemented by large consortia efforts such as ENCODE (44), which also generate significant numbers of haematopoietic TF ChIP-Seq datasets. Collectively, these datasets provide a powerful resource for performing complex integrated analysis and thereby uncovering new insights into the role of TFs in cellular development.

For this paper, we made use of the HAEMCODE ChIP-seq compendium and its large collection of uniformly processed haematopoietic TF ChIP-seq datasets. In contrast to individual projects which provide snapshots of specific developmental time-points, the HAEMCODE compendium integrates large numbers of these snapshots to provide coverage across a wide range of blood cell development. Following identification of pairs of co-occurring motifs, we were therefore able to interrogate co-operative binding events across different cell types. We therefore not only report lists of preferential motif-pairs, but also identify specific motif-pair combinations that are associated with single or subsets of blood cell types. Moreover, we demonstrate that spatially-constrained haematopoietic motif-pairs display elevated levels of sequence conservation, and that two previously unrecognized motif-pairs are critical for full promoter activity when tested by reporter gene assays.

DNA sequence motifs can be represented using a variety of approaches ranging from simple IUPAC strings to position weight matrices, and also more complicated statistical models of TF binding affinities (e.g. dinucleotide weight matrices, K-mers). PWMs are intuitive representations of nucleotide preferences for each base position, involve few parameters and in most cases outperform these alternative models (45). Compared with simple IUPAC strings however, working with PWMs can be complicated by the fact that multiple very similar versions may be present in motif collections such as JASPAR, or be discovered in *de novo* motif discovery applications. Further analysis such as the identification of specific motif-pairs reported here therefore faces the significant problem of many redundant results, where result files are dominated by motif-pairs that consist of virtually identical component motifs. To address this issue, we implemented a new pipeline for PWM similarity and clustering analysis, which enabled us to discover new biologically meaningful and consistent constrained motif-pairs. The use of Jaspar motifs for TF binding site discovery may have excluded pairs involving currently uncharacterised *de novo* motifs or specific preferences in the motif flanking positions, suggesting that future analysis may include those *de novo* motifs without a match to Jaspar motifs. Of note, our current analysis might have recovered some instances of preferential flanking positions not present in Jaspar as new constrained motif-pairs, in line with the notion that an extended motif consensus sequence may indicate the binding of multiprotein complexes.

Despite the rapid increase in the number of published ChIP-seq datasets, the task of correctly identifying functionally significant binding events and predicting transcriptional regulatory mechanisms has proved difficult. Our analysis of all HAEMCODE TF-bound regions revealed that a significant fraction of peaks contained no match to any of the 354 motifs obtained by *de novo* motif discovery of all the individual ChIP-Seq peak lists in HAEMCODE. Furthermore, these ‘motif-less’ peaks predominantly corresponded to regions with only one TF bound. Although it has been reported previously that TF binding can occur in regions without any enriched sequence motifs, we would argue that at least some of these regions may represent false positives identified by the peak calling algorithm. Systematic bias in the form of low complexity regions, repetitive sequences, mis-assembled reference genome and un-annotated regions can contribute to false positive ChIP-seq peaks (46,47). Another plausible explanation for some of the ‘motif-less’ peaks is DNA looping, where regions without motifs were in close vicinity to regions with motifs, and therefore have become cross-linked in the ChIP-Seq protocol. Nevertheless, the observation that ‘motif-less’ peaks are predominantly found in regions only bound by a single TF in the compendium would argue that many of them represent false positives, especially as most TF ChIP-Seq samples so far do not include biological replicates. We would argue therefore that (i) future ChIP-Seq studies should include biological replicates to permit identification of high-confidence binding peaks for each individual TF, and (ii) analysis of existing datasets will be more robust when focussing on peak regions bound by at least two TFs. Nonetheless, our current understanding of ‘functionality’ in relation to TF binding to DNA remains limited (48), and a substantial fraction of reproducible binding events may represent ‘opportunistic’ binding to DNA sequences that happen to be readily accessible.

It is widely accepted that key regulatory TFs function as components of co-operative multimeric complexes sometimes referred to as an enhanceosome. Assembly of multiple TFs and accessory proteins on a given DNA sequence may require precise spacing between the binding sites to facilitate TF interactions on contiguous segments of the DNA. Here, we examined the promoter of the *Cbfa2t3* gene, which encodes a major regulator of blood cell development (35,36). The Cbfa2t3 promoter contains two motif-pairs with preferential spacing—‘E-box + GATA’ (offset 9bp) and ‘Ets + E-box’ (offset 1 bp). Both motif-pairs are present within a 79 bp contiguous stretch, and disrupting the spacing of each motif-pair significantly reduces transcriptional activity. Incorporation of six random nucleotides increases spacing by one half turn of the double helix so that each TF now binds opposing faces of the double helix and thereby allowing us to test the synergistic effect of co-operating TFs. Functional analysis of constrained motif-pairs in two additional haematopoietic promoters not only supported the importance of TF interaction in mediating transcriptional activation, but also validated two previously unknown haematopoietic motif-pairs with constrained spacing, namely the ‘Ets + E-box’ (offset 1bp) and ‘Ets + Homeobox’ (offset 3 bp) motif-pairs. When designing the 6bp insertion, we took into account the binding energies of the bases immediately adjacent to the predicted binding sites by maintaining at least one nucleotide flanking the motifs in the 6bp insertion. In the case of ‘Ets + Homeobox’ and ‘E-box + Gata’ motif-pairs, the spacing is >1 bp and, therefore, an improved design of the inserted random sequence may be considered for future analysis.

Interestingly, our analysis of HAEMCODE datasets revealed that only a small subset of all possible pair-wise combinations of motifs showed evidence for constrained spacing. We found 240 motifs that participated in pairs with preferential spacing, which theoretically could give rise to 28 920 possible combinations of motif-pairs (240 homotypic + 28 680 heterotypic pairs). However, only 2303 motif-pairs were actually found to display preferences for a strict spacing. These observations suggest that a subset of TF combinations is particularly relevant for transcriptional control during haematopoietic development. Preferences for motif-pair combinations also extended to cell type-specificity within the haematopoietic hierarchy in that certain combinations are more prevalent than others in distinct cell types. The seemingly sparse ‘circos’ plot for the dendritic cell type is likely the consequence of small sample numbers for this cell type, which will be improved by increasing the coverage of different TFs in dendritic cell samples. Of note, the HAEMCODE compendium is an on-going effort to add newly published datasets and to increase the coverage across different blood cell types (49). Features of motif-pairs in our dataset also revealed four clusters (Ets, Homeobox, zinc-coordinating, and E-box) that were highly promiscuous and formed many pairs with most (if not all) of the other motif clusters while, in comparison, the remaining 15 motif clusters had smaller numbers of interacting partners. In some ways, this observation is analogous to ‘hubs’ in regulatory networks, and suggests that a subset of ‘hub TFs’ may be able to recruit a wide range of other TFs during the assembly of multimeric TF complexes on DNA.

Binding by multiple TFs to a given region has previously been proposed as a criterion for selecting the most biologically relevant ChIP-seq peaks (50,51). Here, we provide evidence for widespread sequence-specific DNA binding of TF complexes with constrained spacing on haematopoietic gene regulatory elements. Our findings contrast with the recently proposed ‘TF collective’ model (3), where co-occupancy of Drosophila cardiac enhancers was reported not to require specific spacing or orientation of binding sites. Instead, we would argue that both variable and constrained spacing are prevalent in mouse haematopoietic gene regulatory sequences. Moreover, we demonstrate that TF-bound regions containing spatially constrained motif-pairs display elevated sequence conservation both within and across species. This observation is consistent with important transcriptional functions in haematopoietic gene regulation, which we validated using three different promoters and their corresponding motif-spacer mutants in two different cell line models.

Taken together, we highlight specificity of interacting partners, potentially constrained spacing, and cell type-selectivity as important properties of combinatorial transcriptional control processes, with likely roles in the establishment of distinct cell type identities within the haematopoietic system and beyond.

## SUPPLEMENTARY DATA

Supplementary Data are available at NAR Online.

SUPPLEMENTARY DATA
