# Supplementary material for: Constrained transcription factor spacing is prevalent and important for transcriptional control of mouse blood cells
Source: Nucleic Acids Res. 2014 Nov 26;42(22):13513–24. doi: 10.1093/nar/gku1254 (PMC4267662; doi:10.1093/nar/gku1254)
Supplement: SUPPLEMENTARY DATA [file supp_42_22_13513__index.html]

Constrained transcription factor spacing is prevalent and important for transcriptional control of mouse blood cells — Constrained transcription factor spacing is prevalent and important for transcriptional control of mouse blood cells — SUPPLEMENTARY DATA 

# Constrained transcription factor spacing is prevalent and important for transcriptional control of mouse blood cells

## SUPPLEMENTARY DATA

**Files in this Data Supplement:**

- SUPPLEMENTARY DATA
