# Supplementary material for: Constrained transcription factor spacing is prevalent and important for transcriptional control of mouse blood cells
Source: Nucleic Acids Res. 2014 Nov 26;42(22):13513–24. doi: 10.1093/nar/gku1254 (PMC4267662; doi:10.1093/nar/gku1254)

Constrained transcription factor spacing is prevalent and important for transcriptional control of mouse blood cells

Felicia SL Ng[1], Judith Schütte[1], David Ruau[1], Evangelia Diamanti[1], Rebecca Hannah[1], Sarah J Kinston[1], Berthold Göttgens[1*]

[1]Department of Haematology, Wellcome Trust and MRC Cambridge Stem Cell Institute & Cambridge Institute for Medical Research, Cambridge University, Cambridge, CB2 0XY, UK

(Correspondence to: Berthold Göttgens, bg200@cam.ac.uk)

Supplementary Data Table of Contents

Supplementary Methods

Position weight matrices and information content — Page 2
Calculating the detection threshold — Page 3
Mapping ChIP-seq peaks and motif-pair regions to genomic locations — Page 4
PWM similarity and clustering — Page 5
Enrichment of motif pair with significant spacing in ChIP-seq samples — Page 6
GREAT analysis — Page 6
References — Page 7

Supplementary Figures

Figure 1 – Distribution of peak numbers — Page 8
Figure 2 – Motif trimming and offset calculation — Page 9
Figure 3 – DNA sequences for wild-type and mutant promoters of *Atf3*, *Cbfa2t3*, and *Csf3* — Page 10
Figure 4 – Motif-pairs with significant preferential spacing — Page 11
Figure 5 – PWM similarity clustering of the 240 motifs — Page 12
Figure 6 – Heatmap showing the distribution of significant offset values organized by types of motif pairs — Page 13
Figure 7 – Motif-pairs with preferential spacing organized by cell type — Page 14
Figure 8 – Overlapping SNPs/Indels and conservation score analysis — Page 15
Figure 9 – ChIP-seq data supporting the binding of the ‘Ets + E-box’ and ‘E-box + Gata’ motif-pairs on the *Cbfa2t3* promoter — Page 16

Supplementary Tables

Table 1 – ChIP-seq sample details — Page 17
Table 2 – Table of unique motif-pairs with significant preferential spacing — Page 24
Table 3 – Probability matrices of 34 motifs that did not belong to any of the 19 motif clusters — Page 25
Table 4 – GREAT analysis — Page 37
Table 5 – Probability matrices and logos of candidate motif-pairs — Page 41

Supplementary Methods

Position weight matrices and information content. TF DNA-binding preferences or motifs can be represented using position weight matrices (PWM) (1) consisting of 4 rows x $\boldsymbol{w}$ columns for a motif of length $\boldsymbol{w}$ base pairs. PWMs are often derived from experimental data of known TF binding sites and stores nucleotide preferences for each base position. Position ($\boldsymbol{i}$, $\boldsymbol{j}$) in the matrix, denoted as $\boldsymbol{f_{i,j}}$, is the relative frequency of observing (from experimental data, for example) the nucleotide $\boldsymbol{i}$ in position $\boldsymbol{j}$ such that $\sum_{i=1}^{4} f_i = 1$. For DNA-binding motifs, the $\boldsymbol{i}$–th nucleotide represents an element in the array [A, C, G, T] and position $\boldsymbol{j}$ = [1,2,... $\boldsymbol{w}$]. To measure the variability of nucleotide frequencies in a particular position in a binding motif, Schneider (2) proposed an information content based on Shannon's uncertainty to distinguish a binding sequence from a random sequence. The uncertainty of position, $\boldsymbol{j}$, is given by:

$$H_j = -\sum_{i=1}^{4} f_{i,j} \times \log_2(f_{i,j}) \tag{1}$$

And the uncertainty of a randomly sequence position (background model) is given by:

$$H_{bg} = -\sum_{i=1}^{4} p_i \times \log_2(p_i) \tag{2}$$

where $\boldsymbol{p_i}$ denotes the probability of observing nucleotide $\boldsymbol{i}$.

Therefore, the information content (IC) or decrease in uncertainty of position $\boldsymbol{j}$ is defined as:

$$IC_j = H_{bg} - H_j \tag{3}$$

If we assume a uniform background model where $\boldsymbol{p_A}$=$\boldsymbol{p_C}$=$\boldsymbol{p_G}$=$\boldsymbol{p_T}$=0.25, equation 3 becomes:

$$IC_j = \log_2(4) - H_j = 2 + \sum_{i=1}^{4} f_{i,j} \times \log_2(f_{i,j}) \tag{4}$$

Calculating the detection threshold. When scanning ChIP-seq peaks for binding sites, we used a detection threshold for each motif to determine true, high-confidence sites in input sequences. In order to select an appropriate threshold, it is important to understand how HOMER (3) scores a string of DNA sequence, $\boldsymbol{S}$, against a motif PWM, ***motif***. HOMER uses the likelihood ratio test to compare the likelihood that $\boldsymbol{S}$ is drawn from the motif model, ***motif***, to the likelihood that $\boldsymbol{S}$ was drawn from a background model, ***bg***. As an example, consider a test sequence 'GATA' represented in the matrix form, $\boldsymbol{T}$:

$$T = \begin{matrix} A \\ C \\ G \\ T \end{matrix} \begin{bmatrix} 0 & 1 & 0 & 1 \\ 0 & 0 & 0 & 0 \\ 1 & 0 & 0 & 0 \\ 0 & 0 & 1 & 0 \end{bmatrix}$$

Then, the likelihood ratio, $\boldsymbol{d}$, of $\boldsymbol{T}$ being a binding site can be obtained using the formula:

$$d(T) = \log_2 \frac{\mathrm{p}(T|motif)}{\mathrm{p}(T|\mathrm{bg})} = \log_2 \frac{\prod_{j=1}^{w} \prod_{i=1}^{4} f_{i,j}^{T_{i,j}}}{\prod_{j=1}^{w} \prod_{i=1}^{4} p_i^{T_{i,j}}} = \sum_{j=1}^{w} \sum_{i=1}^{4} T_{i,j} \log_2 \frac{f_{i,j}}{p_i} \qquad (5)$$

Using a weight matrix where entries consists of $M_{i,j} = \log_2 \frac{f_{i,j}}{p_i}$ for nucleotide $\boldsymbol{i}$ and position $\boldsymbol{j}$ in the matrix, the score can be obtained from the inner product of the two matrices.

$$d(T) = M^T \cdot T \qquad (6)$$

Assuming a uniform background model and an example motif model, $\boldsymbol{M^T}$, as shown below, the test sequence likelihood score can also be obtained by summing up the individual column log likelihood ratios:

$$d(GATA) = \begin{bmatrix} -4.64 & -4.64 & 1.95 & -4.64 \\ 1.91 & -3.64 & -3.64 & -3.64 \\ -3.64 & -3.64 & -3.64 & 1.91 \\ 1.95 & -4.64 & -4.64 & -4.64 \end{bmatrix} \cdot \begin{bmatrix} 0 & 1 & 0 & 1 \\ 0 & 0 & 0 & 0 \\ 1 & 0 & 0 & 0 \\ 0 & 0 & 1 & 0 \end{bmatrix}$$

$$= \log_2 \frac{f_{G,1}}{0.25} + \log_2 \frac{f_{A,2}}{0.25} + \log_2 \frac{f_{T,3}}{0.25} + \log_2 \frac{f_{A,4}}{0.25}$$

$$= 7.72$$

In this study, we used a stringent detection threshold, $\boldsymbol{D}$, for each Jaspar 'core' motif to identify 'high-confidence' binding sites. Therefore, we calculated $\boldsymbol{D}$ for each 'core' motif by considering only nucleotide positions with high information content (IC ≥ 1) and the maximum probability in each position. Let $f_{max}(j)$be the maximum probability value of column $\boldsymbol{j}$ in a motif model.

$$f_{max}(j) = max\{\{f_i(j)\}_{i=1}^{4}\}\ ;\ \forall\, j = 1 \dots w \quad (7)$$

Then, $\boldsymbol{D}$ is defined as

$$D = \sum_j \log_2 \frac{f_{max}(j)}{0.25}\ , \qquad j = \{\, k{:}\, IC_k \geq 1;\ \forall\, k = 1 \dots w\} \quad (8)$$

Mapping ChIP-seq peaks and motif-pair regions to genomic locations. ChIP-seq peaks containing motif-pairs with significant offset values were mapped to genomic locations using an in-house program that finds overlapping features. Regions were mapped to genomic locations if it overlaps the genomic feature by at least 1bp. First, regions were mapped to promoters using annotation from MPromDb (4). Second, regions were assigned to intragenic and intergenic (within 50kb of a gene) locations based on annotation from UCSC (downloaded using Table Browser). If no gene was found within that range, motif-pairs were assigned to gene 'deserts'.

For motif-pair regions, we examined the overlap with promoters, 5'UTR, 3'UTR, introns, first exon, later exons and known enhancers using an in-house program. Coordinates for UTRs, introns and exons were obtained from UCSC's Table Browser and the collection of known human and mouse enhancers was obtained from the Vista enhancer browser: http://enhancer.lbl.gov/) (5). Both human (hg19) and mouse (mm9) enhancer datasets were lifted over with the liftOver tool (https://genome.ucsc.edu/util.html) to mm10 before performing the mapping.

PWM similarity and clustering. To cluster a collection of PWMs, a 2-dimensional distance score matrix was calculated for all pair-wise comparison of motifs. In this study, a distance score matrix, $\boldsymbol{M}$, was calculated for 240 Jaspar PWMs (motifs participating in a pair with significant offset) where $\boldsymbol{M}$ is a matrix of size 240 x 240. Position ($\boldsymbol{a}$, $\boldsymbol{b}$) in $\boldsymbol{M}$ contains the mean Euclidean distance of all overlapping columns of a pair of PWMs – $\boldsymbol{A}$ and $\boldsymbol{B}$, where $\boldsymbol{a}$=[1, 2,…, 240] and $\boldsymbol{b}$=[1, 2,…, 240]. To illustrate this, the diagram below shows an example of two PWMs, $\boldsymbol{A}$ and $\boldsymbol{B}$, with four overlapping columns. A vector, $\boldsymbol{s} = [s_1, s_2, \cdots s_j]$, can then be calculated for each column, $\boldsymbol{j}$, by taking the Euclidean distance of two vectors $\boldsymbol{A_{1:4,j}}$ and $\boldsymbol{B_{1:4,j}}$ for $\boldsymbol{j}$=[1,2,3,4]. Finally, $\boldsymbol{M_{a,b}}$ is the mean of the vector $\boldsymbol{s}$.

$$A = \begin{matrix} A \\ C \\ G \\ T \end{matrix} \begin{bmatrix} A_{1,1} & A_{1,2} & A_{1,3} & A_{1,4} \\ A_{2,1} & A_{2,2} & A_{2,3} & A_{2,4} \\ A_{3,1} & A_{3,2} & A_{3,3} & A_{3,4} \\ A_{4,1} & A_{4,2} & A_{4,3} & A_{4,4} \end{bmatrix}$$

$$B = \begin{matrix} A \\ C \\ G \\ T \end{matrix} \begin{bmatrix} B_{1,1} & B_{1,2} & B_{1,3} & B_{1,4} \\ B_{2,1} & B_{2,2} & B_{2,3} & B_{2,4} \\ B_{3,1} & B_{3,2} & B_{3,3} & B_{3,4} \\ B_{4,1} & B_{4,2} & B_{4,3} & B_{4,4} \end{bmatrix}$$

$$s = [s_1 \quad s_2 \quad s_3 \quad s_4]$$

There are many ways to align the columns of two PWMs but the objective is to find the best alignment that will yield the best (smallest) distance score. One way is to compute $\boldsymbol{s}$ for all possible column alignments but this is rather inefficient. We chose to do this by pair-wise alignment of the IUPAC sequence of the PWMs to speed up the computation. For each $\boldsymbol{M_{a,b}}$, the query motif, $\boldsymbol{q}$, is aligned to the target motif, $\boldsymbol{t}$, using local (Smith Waterman) alignment (6) (match =3, mismatch =-1, gap opening = -1, gap extension = -0.5). If there is a minimum of 3bp overlap, we calculate the Euclidean distance between the shorter PWM and the overlapping segments of the longer motif. Otherwise, the score is set to the maximum distance (i.e. $\sqrt{2}$). For any pair of motifs, the best similarity score is kept for the alignments of $\boldsymbol{q}$ against both orientations of $\boldsymbol{t}$ (original and reverse complement). Because the Euclidean

distance for '$\boldsymbol{A}$ vs $\boldsymbol{B}$' is the same as '$\boldsymbol{B}$ vs $\boldsymbol{A}$', we only calculate the upper triangle of $\boldsymbol{M}$. R source code for the PWM similarity clustering pipeline is available on bitbucket (https://bitbucket.org/feliciang/publication-motif-pair).

Enrichment of motif-pair with significant spacing in ChIP-seq samples. Hypergeometric p-values were computed in R to assess enrichment of motif-pairs in ChIP-seq peak regions of a particular sample (equation 9). For each motif pair (with a total of $\boldsymbol{n}$ regions), the hypergeometric test calculates the probability of observing $\boldsymbol{k}$ motif-pair regions overlapping the peaks in a ChIP-seq sample with a total of $\boldsymbol{K}$ peaks. The regions in the test can be illustrated in a contingency table as shown below. Here, $\boldsymbol{N}$ denotes the total number of peak regions from all 289 HAEMCODE samples (overlapping regions merged) and contains $\boldsymbol{K}$ peak regions and $\boldsymbol{N}$-$\boldsymbol{K}$ non-peak regions.

$$P(X = k) = \frac{\binom{K}{k}\binom{N-K}{n-k}}{\binom{N}{n}} \tag{9}$$

	motif-pair	not motif-pair	
peak	$\boldsymbol{k}$	$\boldsymbol{K - k}$	$\boldsymbol{K}$
not a peak	$\boldsymbol{n - k}$	$\boldsymbol{N + k - n - K}$	$\boldsymbol{N - K}$
	$\boldsymbol{n}$	$\boldsymbol{N - n}$	$\boldsymbol{N}$

GREAT analysis. Genomic coordinates were obtained for each candidate motif-pair by combining all unique regions of the specified spacing. The coordinates were then lifted over to mm9 genome using the liftOver tool (https://genome.ucsc.edu/util.html) and used as input for the GREAT analysis with default settings (http://bejerano.stanford.edu/great/public/html/) (GREAT version 2.0.2) (7). Significant results (pval ≤ 0.05) were summarized in Supplementary Table 4.

References

1. Stormo, G.D., Schneider, T.D., Gold, L. and Ehrenfeucht, A. (1982) Use of the 'Perceptron' algorithm to distinguish translational initiation sites in E. coli. *Nucleic acids research*, **10**, 2997-3011.
2. Schneider, T.D., Stormo, G.D., Gold, L. and Ehrenfeucht, A. (1986) Information content of binding sites on nucleotide sequences. *Journal of molecular biology*, **188**, 415-431.
3. Heinz, S., Benner, C., Spann, N., Bertolino, E., Lin, Y.C., Laslo, P., Cheng, J.X., Murre, C., Singh, H. and Glass, C.K. (2010) Simple combinations of lineage-determining transcription factors prime cis-regulatory elements required for macrophage and B cell identities. *Molecular cell*, **38**, 576-589.
4. Gupta, R., Bhattacharyya, A., Agosto-Perez, F.J., Wickramasinghe, P. and Davuluri, R.V. (2011) MPromDb update 2010: an integrated resource for annotation and visualization of mammalian gene promoters and ChIP-seq experimental data. *Nucleic acids research*, **39**, D92-97.
5. Visel, A., Minovitsky, S., Dubchak, I. and Pennacchio, L.A. (2007) VISTA Enhancer Browser--a database of tissue-specific human enhancers. *Nucleic acids research*, **35**, D88-92.
6. Smith, T.F. and Waterman, M.S. (1981) Identification of common molecular subsequences. *Journal of molecular biology*, **147**, 195-197.
7. McLean, C.Y., Bristor, D., Hiller, M., Clarke, S.L., Schaar, B.T., Lowe, C.B., Wenger, A.M. and Bejerano, G. (2010) GREAT improves functional interpretation of cis-regulatory regions. *Nature biotechnology*, **28**, 495-501.

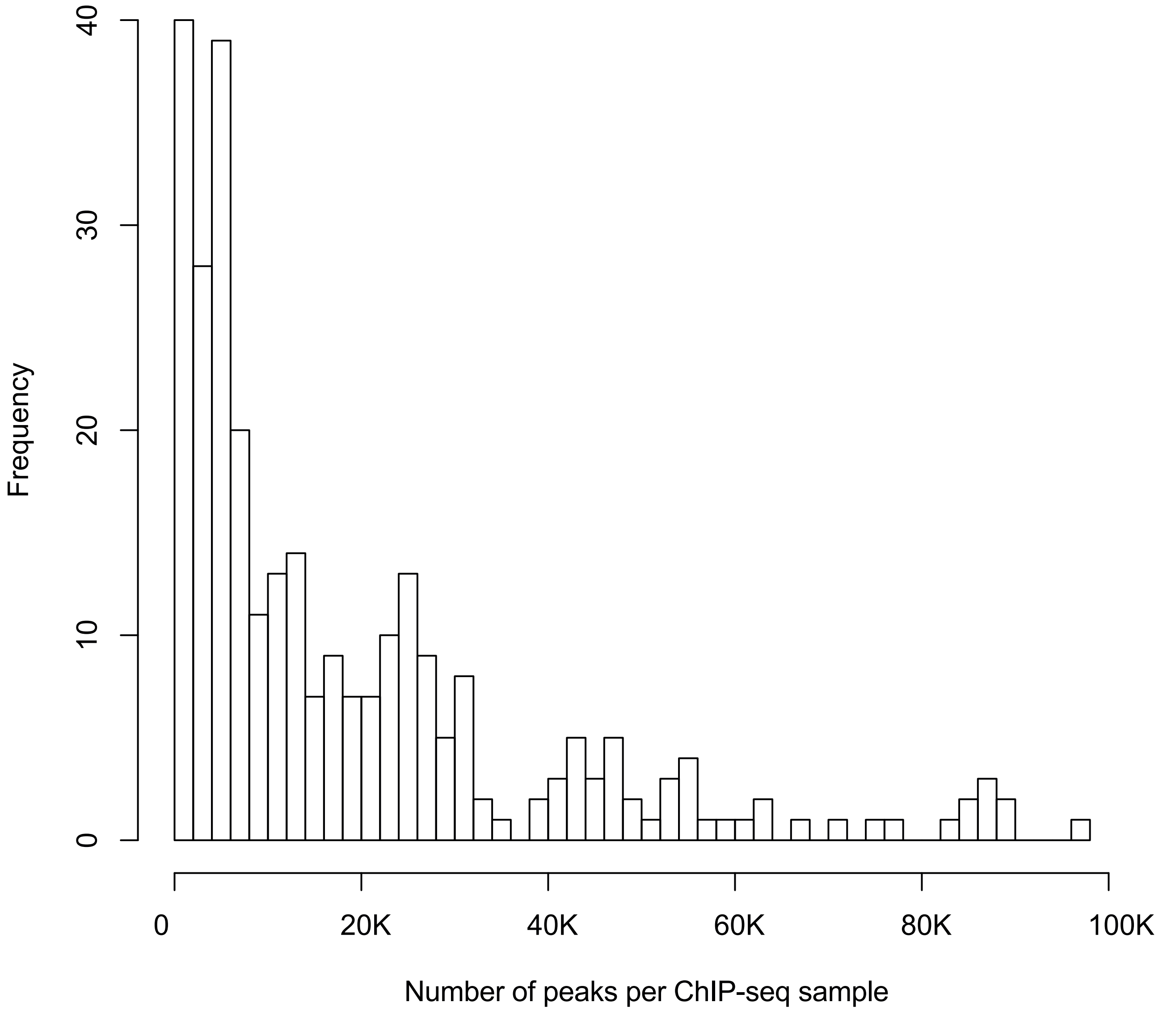

Supplementary Figure 1. Distribution of peak numbers. Histogram of the number of peaks per sample for the 289 HAEMCODE samples.

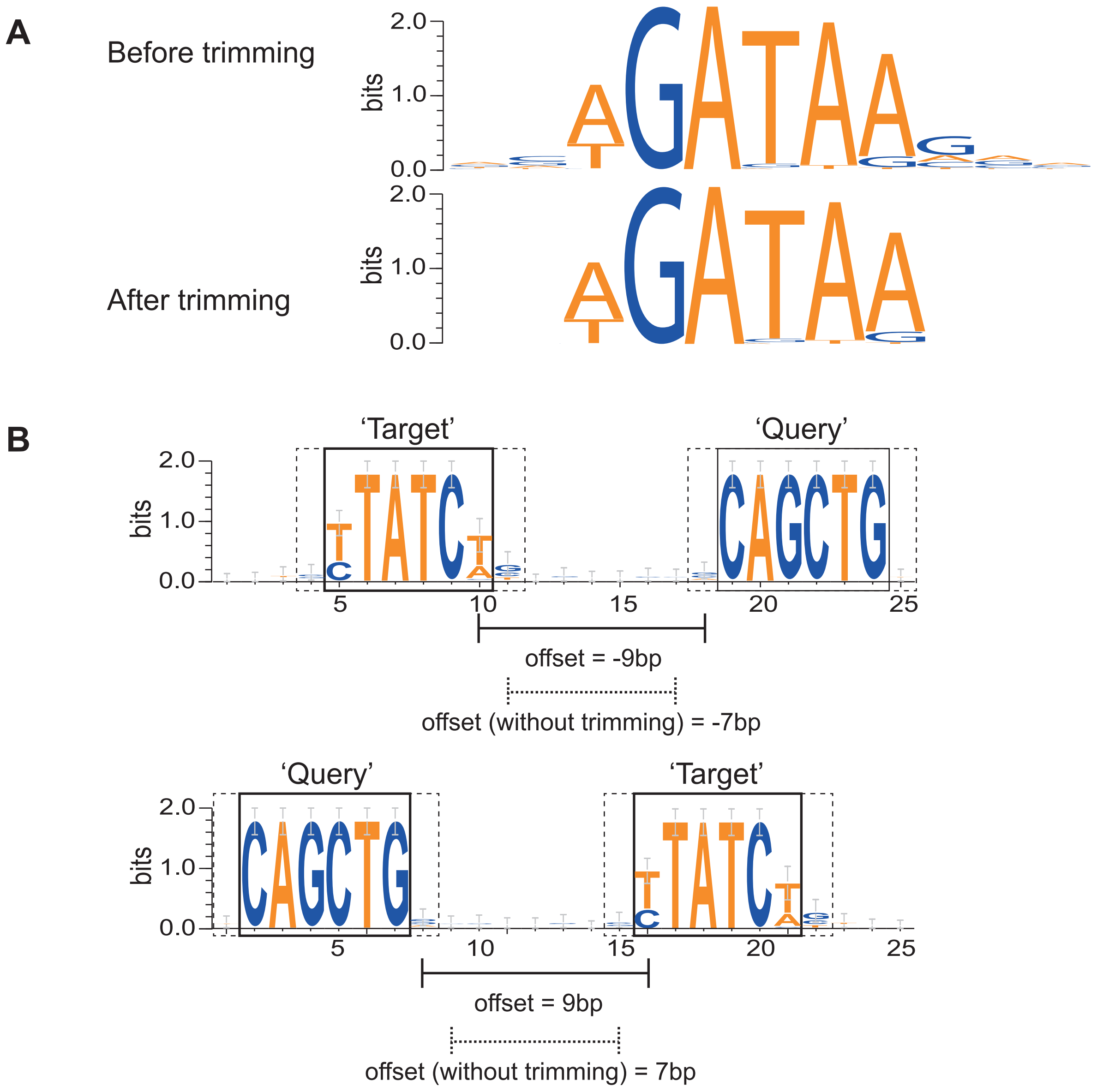

Supplementary Figure 2. Motif trimming and offset calculation. (A) Example of a motif before and after trimming of PWM columns with information content less than 0.5. (B) Example of how offset values were calculated for a pair of motifs – 'query' and 'target'. Offset values are edge-to-edge distance values and were calculated from the first nucleotide adjacent to the 'query' motif to the first nucleotide of the 'target' motif. If the 'query' motif lies downstream of the 'target' motif, a negative value is assigned. Otherwise, the offset value is positive. Without motif trimming, flanking regions that may represent 'true' spacing may affect offset value calculations.

Atf3 (chr1:191183232-191183778, mm10)

Wild-type:

```
GCTAGCTTTTCCTGAAGTTTTAGAAAAATGACCACACATTTTAGAGAAAGGTCGTGCCCGCTTCCCAGCCTCACC
TAGTCTGGGCCACTGCCAGGATCCGCCTCCCAGCACCACCACCCCCCCTTCCCCAACCTAGCGGAGGGAGAGATG
CCAGGGCGGTGGAGTCATGCCGCTGGCTTGGGCACCATTGGCTCATGCCTGGAACACGCAGCGGCGAATACGCAC
ATCTGGCGGTGCGCCCGGGCGGCTCCCAGCCTGATAAGGAGATAGAGGGCGGGCGGGTGTGTGTGTCCCGGGCGT
GCGAGGAACGCAGGACGCGCCGTGACCGCCCCTTCTCGCACTTGCATCACCAAGTCCGCGGTCTCCACCCACCTT
TTGCCCCGCCCCCGTCCCTCCTTCACCCCTCCTCTGGCCAGTTCTCCCTGGAAGCTATTAATAGCATTACGTCAG
CCTGGGATTGGTAACCTGGAGTTAAGCGGGCTCCCTGCCAACGCGAGGGCTTTAAAAGGGGTGATGCAACGCGCT
CCCAGCCACAGTCTCACTCAGCGAGACGAAGCTT
```

Ets+Hox: … TCTGGCCAGTTCTCCCTGGAAGTGAGGGCTATTAATAGCATTACGTCAGC…

Cbfa2t3 (chr8:122699013-122699357, mm10)

Wild-type:

```
GGTACCGAGCTCTTACGCGTGCTAGCAGGCGGGATAGGAGGAAGTTGTTGGGAAGTCAGACCGGAATGGCATGGT
GGAGGGAGAACCGGCAACCAGGCAGATGGTTCCTGACGAGGAAGCTCTGGGCACAGCTGCAGGCCCCCGACCCCC
ACCGCATTATCACTGTGACACAGCTGGCTGCCTCACCCCTGAAGGCTGCAGGAGGACCTCCCCCATGCTGTCCCC
AAGCCCGCCCCGTGTCACATGAGGCCCTGCAGACTCCCACCCTCCGTCCAGGGCCACAACCCAGCTCTGCCGGCT
GTAGTGACTAGAAAGGCCTGGAGCCTCCAAGGAACAGAGGCACGGGCTCCGAGACGCCAAAGCTCCTCCAGAAGC
TTGGCATTCCGGTACTGTTGGT
```

E-box+Gata: ..CCCCCACCGCATTATCAGACTCGCTGTGACACAGCTGGCTGCCTCACCCC…

E-box+Ets: ..AGAACCGGCAACCAGGCAGATGGTAGGTGTTCCTGACGAGGAAGCTCTGG…

Csf3 (chr11:98701062-98701370, mm10)

Wild-type:

```
GGTACCGAGCTCTTACGCGTGCTAGCGATCCAACACCCTGCAGCGATTCAGGCCTGGGTGTGGCTGGAAGAGAGG
AAGAGAGTTTGAGGGGGGGACAAGACGTCAAGGAAGGAACAGAGATTCCCCGATTTCACAAAAACTTTCGCAAAC
AGCTTTTCCCCAACCCCCTGCATTGTCCTGAGCTATCATCAAATTTGCATAAATCCTGGGAAGTTATTACTAAGC
CTGAGTTGCCCCAGCCCCAGGTAATTTCCTCCCGGGGCCTTGATGGCTTTATGTATAAAGGCCCCCTGGAGCTGG
GCCCTGGCAGAGCCCAGAGCTGCAGCCCAGATCACAAGCTTGGCATTCCGGTACTGTTGGT
```

Ets+Hox: … ATTTGCATAAATCCTGGGAAGTGCCCGTTATTACTAAGCCTGAGTTGCC…

Supplementary Figure 3. DNA sequences for wild-type and mutant promoters of Atf3, Cbfa2t3 and Csf3. All promoters were cloned into pGL2 basic (Promega) digested with NheI and HindIII (restriction enzymes from NEB, marked in red). The sequences for the Atf3 promoter was generated by GeneArt® Gene Synthesis and the sequences for the Cbfa2t3 and Csf3 promoters were generated by GeneArt® Strings from Life Technologies. The Atf3 promoter was inserted into pGL2 basic by traditional cloning using restriction enzymes. The Cbfa2t3 and Csf3 promoters were cloned by Gibson Assembly. The 20bp overlapping ends are marked in grey. The DNA binding motifs of interest are highlighted as follows: Ets = pink, Homeobox (Hox) = light green, E-box = blue, Gata = dark green. The nucleotides that were introduced to disrupt the spacing between two DNA binding motifs are underlined and in bold.

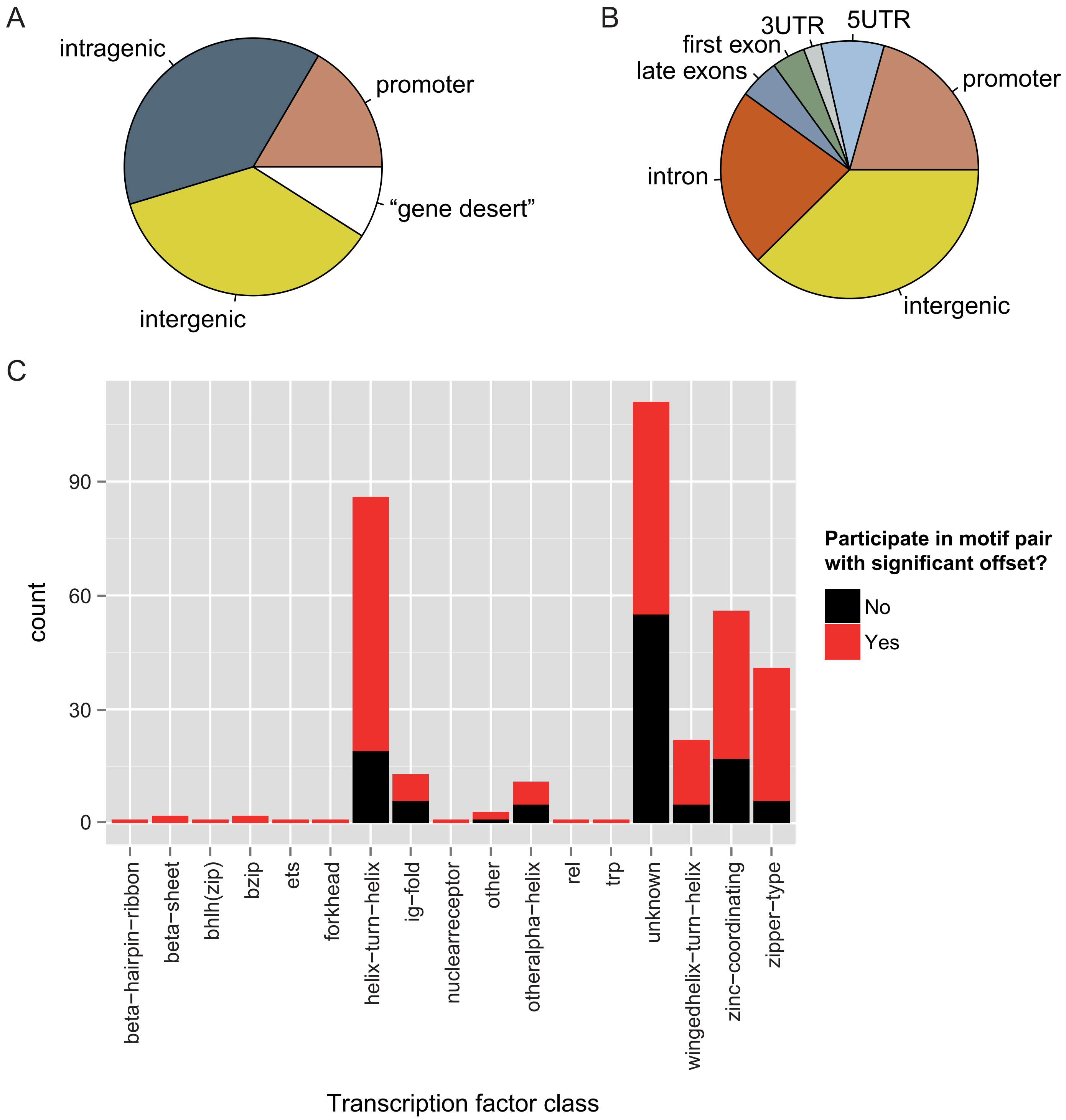

Supplementary Figure 4. Motif-pairs with significant preferential spacing. (A) TF-bound regions (ChIP-seq peaks) containing motif-pairs mapped to genomic locations. (B) Motif-pair regions mapped to genomic locations. See supplementary methods (‘Mapping ChIP-seq peaks and motif-pair regions to genomic locations’) for mapping procedure. (C) TF classes (from Jaspar annotation) of all 354 motifs tested, red bars indicate motifs that participate in motif-pairs with significant offset values.

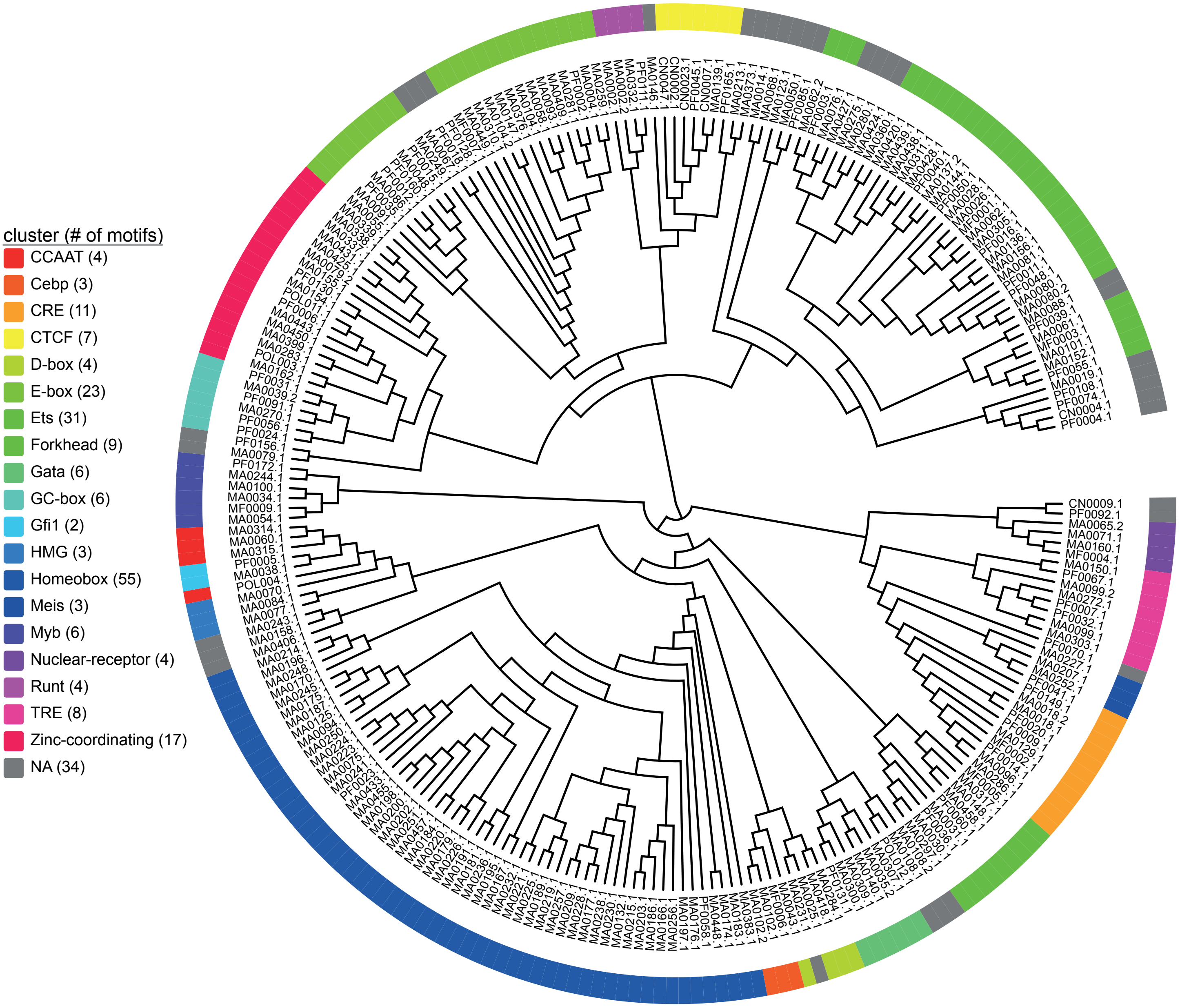

Supplementary Figure 5. PWM similarity clustering of the 240 motifs that participate in a motif-pair with significant spacing. Motifs were grouped into clusters of similar binding sequence motifs and the circular cladogram displays the extent of similarity amongst all motifs. The colour bar around the cladogram indicates the clusters in which each motif belongs to. Total number of motifs in each cluster is shown in the figure key.

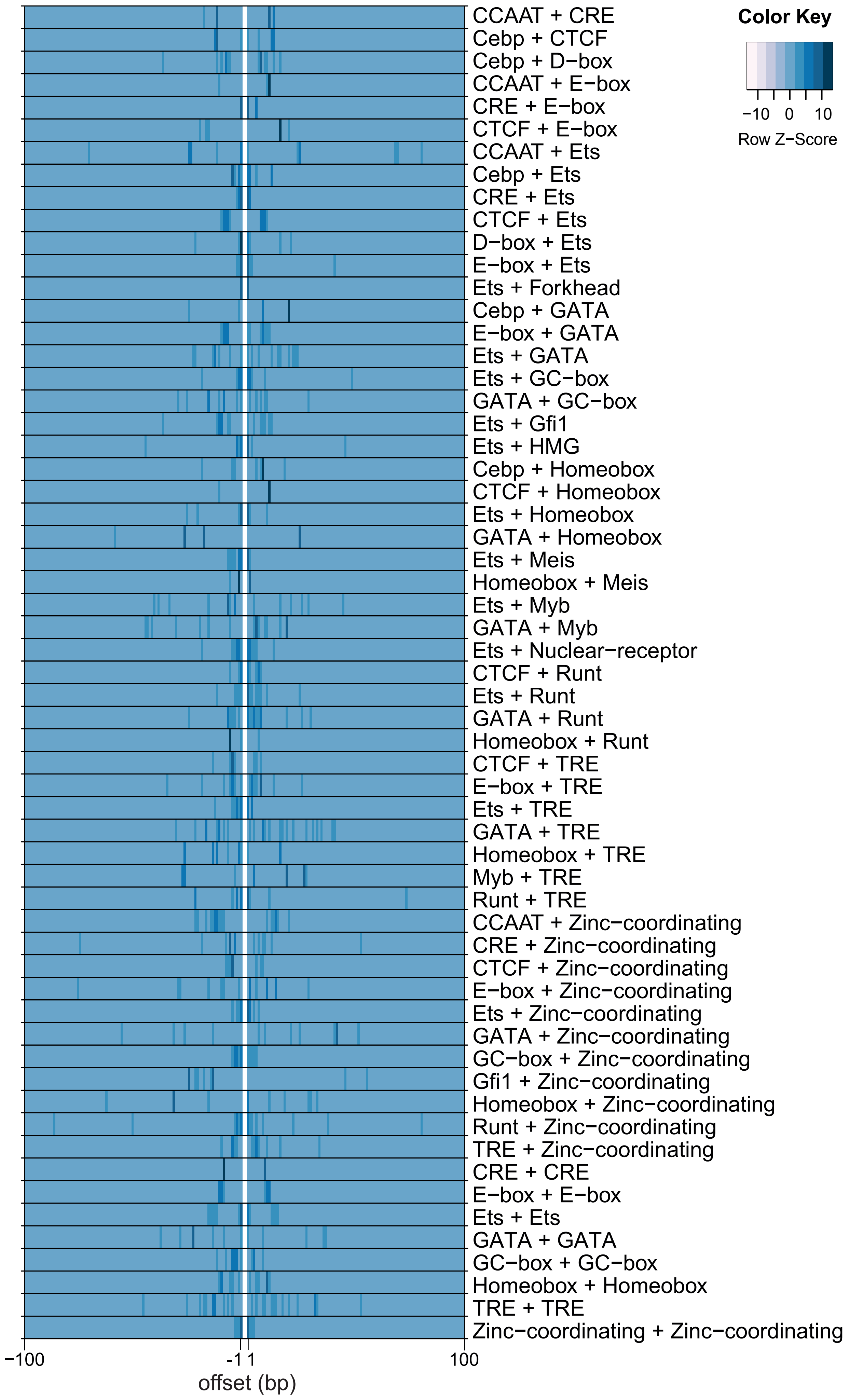

Supplementary Figure 6. Heatmap showing the distribution of significant offset values organized by types of motif-pairs. The x-axis shows the offset values tested [-100,…, -1, +1,…, +100] and the y-axis shows the motif-pairs organized by motif clusters. Colours at each offset value for a particular row in the heatmap denote the number of unique motif-pairs with that offset value (dark blue - high, light blue - low).

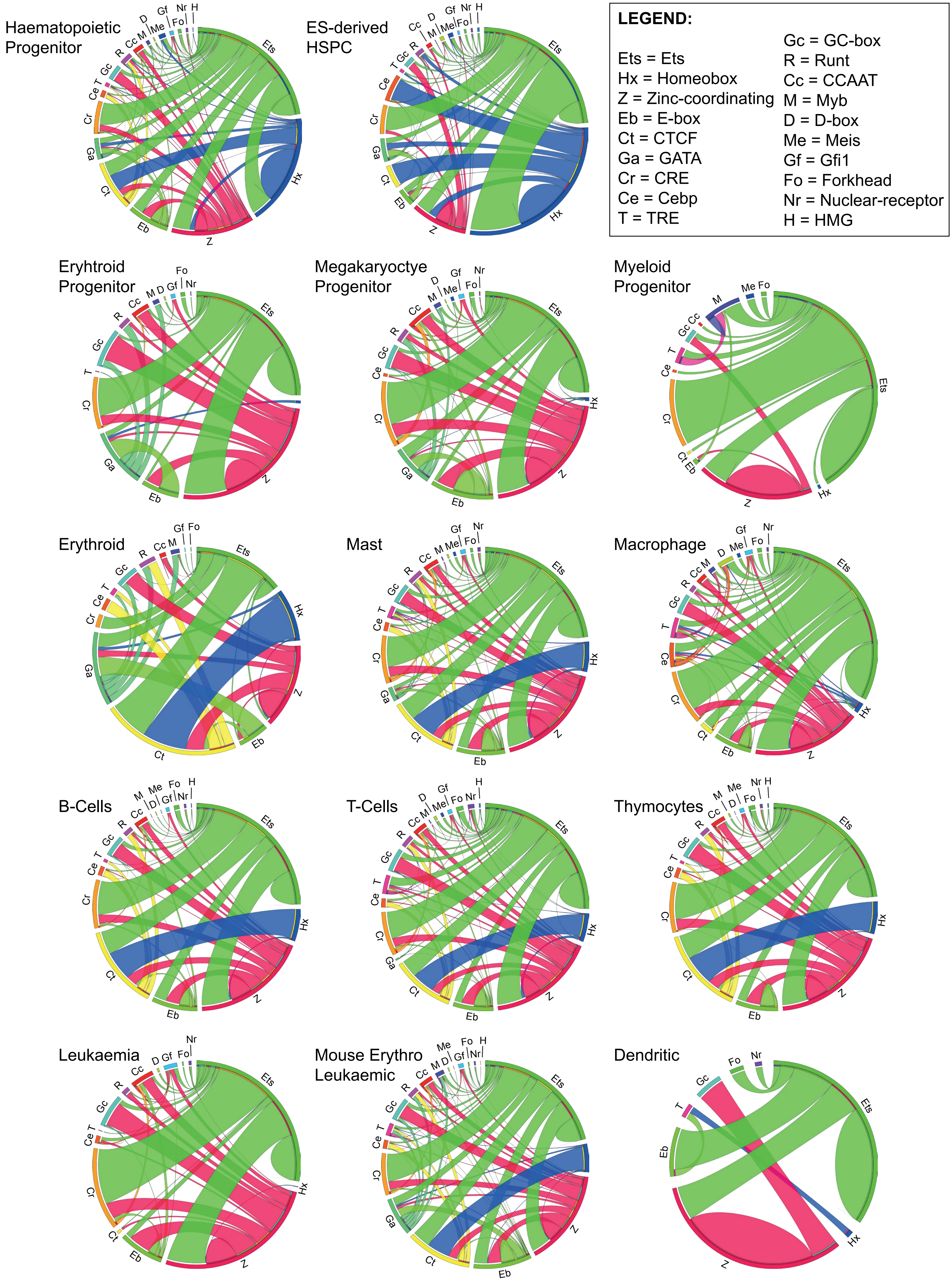

Supplementary Figure 7. Motif-pairs with preferential spacing organized by cell type. Each 'circos' plot shows the abundance of different types of motif-pairs in each cell type. Clusters were defined by PWM similarity clustering (Supplementary Figure 5) and ribbon thickness is proportional to the number of unique motif-pairs in each cell type (multiple offset values not counted).

A

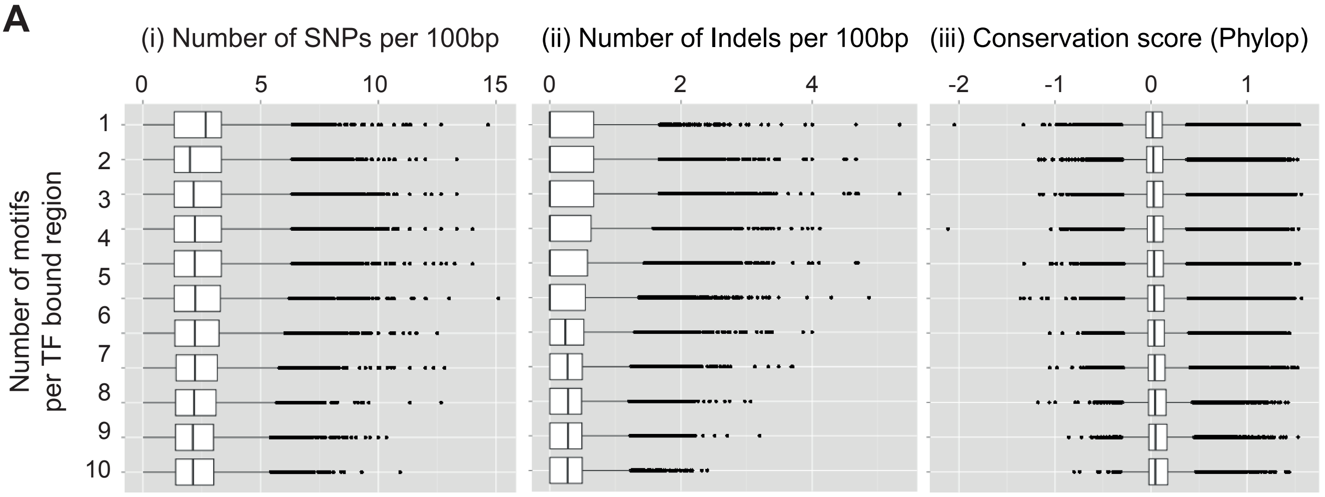

B

	SNP	Indel	PhyloP
**0 vs 1 or more motif-pairs**	D=0.1025, p-val < 2e-16	D=0.1743, p-val < 2e-16	D=0.061, p-val < 2e-16
**0 vs 2 or more motif-pairs**	D=0.2176, p-val < 2e-16	D=0.3053, p-val < 2e-16	D=0.1525, p-val < 2e-16

Supplementary Figure 8. Overlapping SNPs/Indels and conservation score analysis. (A) Boxplot showing the distribution of the number of (i) SNPs or (ii) Indels or (iii) conservation scores (PhyloP) in TF-bound regions (ChIP-seq peaks). The y-axis shows the number of motifs (i.e. predicted binding sites) per TF-bound region. (B) Kolmogorov-smirnov test was performed in R to compare the distribution of SNP, Indel or PhyloP values in peak regions with 0 motif-pairs against peak regions with ≥1 motif-pairs and ≥2 motif-pairs. A significant p-value indicate the two distributions are significantly different and D is the K-S test statistic.

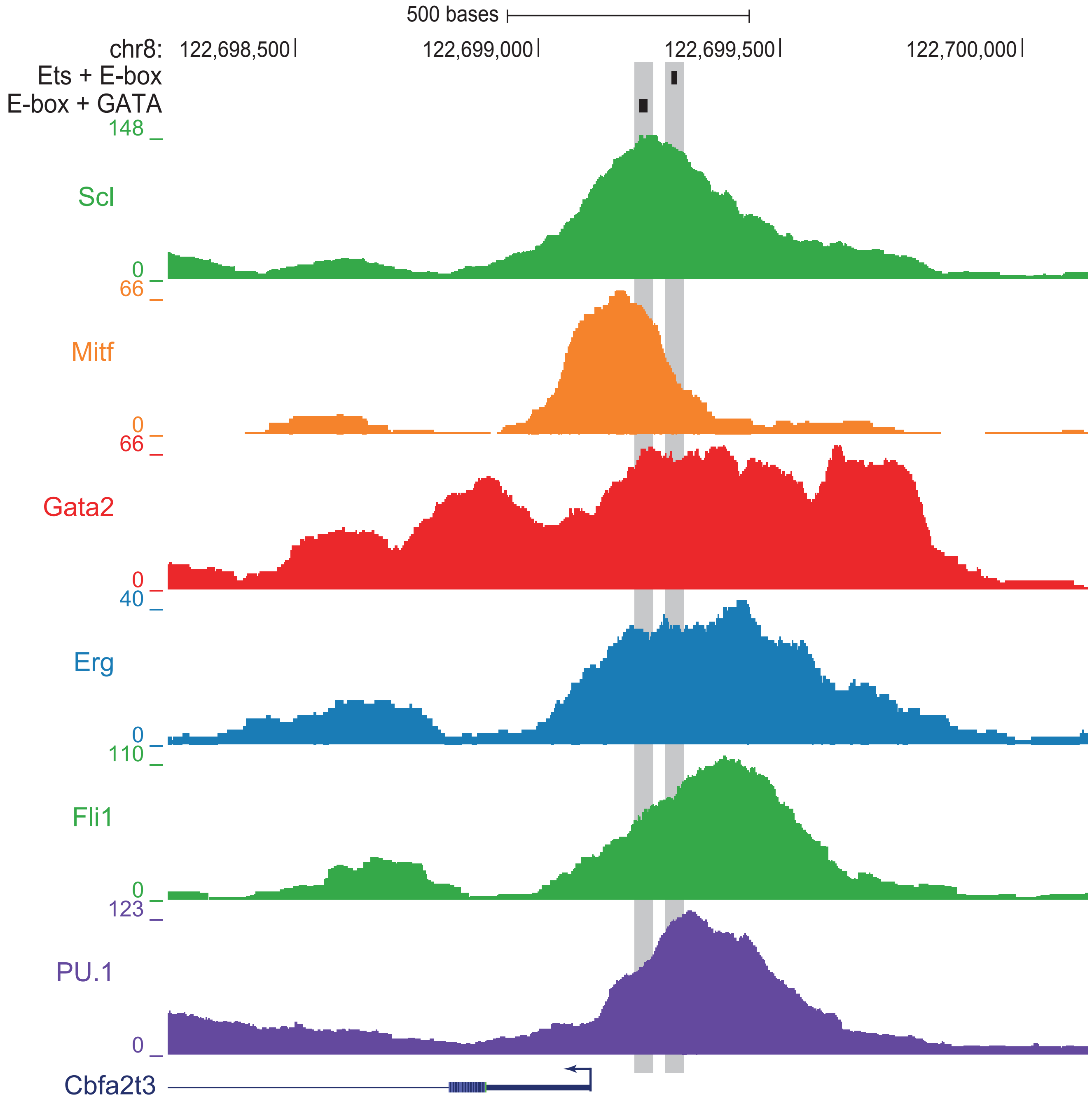

Supplementary Figure 9. ChIP-seq data supporting the binding of ‘Ets + E-box’ and ‘E-box + Gata’ motif-pairs on the Cbfa2t3 promoter. The first two tracks shows motif-pair regions for the motif-pairs ‘Ets + E-box’ and ‘E-box + Gata’ (black rectabgles). The remaining tracks shows ChIP-seq binding sites for the E-box TFs (Scl and Mitf), GATA TF (Gata2), and Ets TFs (Erg, Fli1, and PU.1) in primary mast cells (GSE48046).

Supplementary Table 1. ChIP-seq sample details. Sample details include accession numbers, transcription factor names and cell types assigned based on annotation from the source database (NCBI GEO or EMBL-EBI ENA).

(A) Details of the 289 samples used in this study.

transcription_factor	cell_type	GSE	GSM
Cbfa2t3	Mouse Erythro Leukaemic	ERA000161	ERX002124
Cbfa2t3	Mouse Erythro Leukaemic	ERA000161	ERX002126
Gata1	Mouse Erythro Leukaemic	ERA000161	ERX002128
Gata1	Mouse Erythro Leukaemic	ERA000161	ERX002130
Ldb1	Mouse Erythro Leukaemic	ERA000161	ERX002132
Ldb1	Mouse Erythro Leukaemic	ERA000161	ERX002134
Tal1	Mouse Erythro Leukaemic	ERA000161	ERX002136
Tal1	Mouse Erythro Leukaemic	ERA000161	ERX002138
Cbfa2t2	Mouse Erythro Leukaemic	ERA000161	ERX002140
Cbfa2t2	Mouse Erythro Leukaemic	ERA000161	ERX002142
Gata1	Erythroid	ERA195911	ERX206185-ERX206187
Gata1	Erythroid	ERA195911	ERX206186-ERX206189
Rela	Macrophages	GSE16723	GSM611116-GSM611117
Gata1	Erythroid	GSE18164	GSM453997
Tal1	Erythroid	GSE18720	GSM464634
Tal1	Erythroid	GSE18720	GSM464636
Ebf1	B-Cells	GSE19971	GSM499030
Gata3	T-Cells	GSE20898	GSM523221
Gata3	T-Cells	GSE20898	GSM523222
Gata3	T-Cells	GSE20898	GSM523223
Gata3	T-Cells	GSE20898	GSM523224
Gata3	T-Cells	GSE20898	GSM523225
Gata3	T-Cells	GSE20898	GSM523226
Gata3	T-Cells	GSE20898	GSM523228
Gata3	T-Cells	GSE20898	GSM523229
Gata3	T-Cells	GSE20898	GSM523230
Gata3	T-Cells	GSE20898	GSM523231
Fli1	T-Cells	GSE20898	GSM654874
Rag2	Thymocytes	GSE21207	GSM530318
Sfpi1	Macrophages	GSE21512	GSM537983
Cebpa	Macrophages	GSE21512	GSM537984
Cebpb	Macrophages	GSE21512	GSM537985
Sfpi1	B-Cells	GSE21512	GSM537989
Pou2f2	B-Cells	GSE21512	GSM537990
Sfpi1	B-Cells	GSE21614	GSM539537-GSM539538
Smad3	B-Cells	GSE21614	GSM539545
pStat3	T-Cells	GSE21669	GSM540722

Sfpi1	Erythroid Progenitors	GSE21953	GSM545882-GSM545885
Sfpi1	Erythroid Progenitors	GSE21953	GSM545888-GSM545891
Foxo1	B-Cells	GSE21978	GSM546525
Ctcf	B-Cells	GSE21978	GSM546526
Myb	Myeloid Progenitors	GSE22095	GSM549337-GSM549339-GSM549341
Stat4	T-Cells	GSE22104	GSM550303
Stat6	T-Cells	GSE22104	GSM550311
Fli1	Haematopoietic Progenitors	GSE22178	GSM552233
Gata2	Haematopoietic Progenitors	GSE22178	GSM552234
Gfi1b	Haematopoietic Progenitors	GSE22178	GSM552235
Lmo2	Haematopoietic Progenitors	GSE22178	GSM552237
Lyl1	Haematopoietic Progenitors	GSE22178	GSM552238
Meis1	Haematopoietic Progenitors	GSE22178	GSM552239
Sfpi1	Haematopoietic Progenitors	GSE22178	GSM552240
Runx1	Haematopoietic Progenitors	GSE22178	GSM552241
Tal1	Haematopoietic Progenitors	GSE22178	GSM552242
Ldb1	Haematopoietic Progenitors	GSE26031	GSM641909
Tal1	Haematopoietic Progenitors	GSE26031	GSM641910
Gata2	Haematopoietic Progenitors	GSE26031	GSM641911
Ctcf	B-Cells	GSE26257	GSM644975
Rad21	B-Cells	GSE26257	GSM644976
Stat3	T-Cells	GSE26552	GSM652877
Stat5a/b	T-Cells	GSE26552	GSM652878
Stat3	Dendritic	GSE27161	GSM671415
Stat3	Dendritic	GSE27161	GSM671416
Stat5b	Dendritic	GSE27161	GSM671418
Stat5b	Dendritic	GSE27161	GSM671419
Mllt3	Leukaemia	GSE29130	GSM721212
Gata2	Erythroid Progenitors	GSE29193	GSM722387
Smad1	Erythroid Progenitors	GSE29193	GSM722388
Gata1	Erythroid Progenitors	GSE29193	GSM722390
Smad1	Erythroid Progenitors	GSE29193	GSM722391
Notch1	Leukaemia	GSE29600	GSM732913
Rbpj	Leukaemia	GSE29600	GSM732914
Notch1	Leukaemia	GSE29600	GSM732916
Rbpj	Leukaemia	GSE29600	GSM732917
Tal1	Erythroid	GSE30142	GSM746555-GSM746556
Gata1	Erythroid	GSE30142	GSM746568
Tal1	Erythroid	GSE30142	GSM746571-GSM746572
Gata1	Erythroid	GSE30142	GSM746581-GSM746582
Tal1	Erythroid	GSE30142	GSM746583-GSM746584
Tcf7	Haematopoietic Progenitors	GSE31221	GSM773994
Runx1	Haematopoietic Progenitors	GSE31221	GSM773995-GSM773996
Sfpi1	T-Cells	GSE31235	GSM774291
Sfpi1	T-Cells	GSE31235	GSM774292

Sfpi1	T-Cells	GSE31235	GSM774293
Gata3	T-Cells	GSE31235	GSM774295
Gata3	T-Cells	GSE31235	GSM774296
Gata3	T-Cells	GSE31235	GSM774297
Gata2	Megakaryocyte Progenitors	GSE31331	GSM777091
Gata1	Megakaryocyte Progenitors	GSE31331	GSM777092
Ets1	Megakaryocyte Progenitors	GSE31331	GSM777093
Gfi1	Leukaemia	GSE31657	GSM786037
Meis1	Myeloblastic	GSE33518	GSM842248-GSM842251
Hoxa	Myeloblastic	GSE33518	GSM842249-GSM842252
Runx1	Megakaryocyte Progenitors	GSE33653	GSM832311
Cbfb	Megakaryocyte Progenitors	GSE33653	GSM832312
Rnf2	Megakaryocyte Progenitors	GSE33653	GSM832313
Runx1	Megakaryocyte Progenitors	GSE33653	GSM832315
Cbfb	Megakaryocyte Progenitors	GSE33653	GSM832316
Rnf2	Megakaryocyte Progenitors	GSE33653	GSM832317
Runx1	Megakaryocyte Progenitors	GSE33653	GSM832319
Rnf2	Megakaryocyte Progenitors	GSE33653	GSM832320
Runx1	Thymocytes	GSE33653	GSM832321
Cbfb	Thymocytes	GSE33653	GSM832322
Rnf2	Thymocytes	GSE33653	GSM832323
Tbx21	T-Cells	GSE33802	GSM836124
Stat1	Macrophages	GSE33913	GSM838681
Stat1	Macrophages	GSE33913	GSM838683
Stat1	Macrophages	GSE33913	GSM838685
Stat1	Macrophages	GSE33913	GSM838687
Stat1	Macrophages	GSE33913	GSM838689
Stat1	Macrophages	GSE33913	GSM838691
Hoxb4	ES-derived HSPC	GSE34014	GSM840466
Hoxb4	ES-derived HSPC	GSE34014	GSM840468
Hoxb4	ES-derived HSPC	GSE34014	GSM840470
Mllt3	Leukaemia	GSE34261	GSM846042
Notch1	Leukaemia	GSE34954	GSM859512
Gata1 (V205G)	Megakaryocyte Progenitors	GSE35644	GSM872894
Ebf1	B-Cells	GSE35857	GSM876622-GSM876623
Ebf1	B-Cells	GSE35857	GSM876624-GSM876625
Ctcf	Thymocytes	GSE36027	GSM918734
Ctcf	Mouse Erythro Leukaemic	GSE36027	GSM918744
Ctcf	Erythroid	GSE36029	GSM923570
Ctcf	Erythroid	GSE36029	GSM923571
Gata1	Erythroid	GSE36029	GSM923572
Ctcf	Mouse Erythro Leukaemic	GSE36029	GSM923573
Gata1	Erythroid	GSE36029	GSM923575
Tal1	Erythroid	GSE36029	GSM923576
Tal1	Mouse Erythro Leukaemic	GSE36029	GSM923578
Tal1	Erythroid	GSE36029	GSM923579

Gata1	Megakaryocyte Progenitors	GSE36029	GSM923586
Ep300	Mouse Erythro Leukaemic	GSE36030	GSM912893
Ctcf	Mouse Erythro Leukaemic	GSE36030	GSM912896
Mafk	Mouse Erythro Leukaemic	GSE36030	GSM912899
Myb	Mouse Erythro Leukaemic	GSE36030	GSM912903
Gata1	Mouse Erythro Leukaemic	GSE36030	GSM912907
Jund	Mouse Erythro Leukaemic	GSE36030	GSM912915
Max	Mouse Erythro Leukaemic	GSE36030	GSM912919
Ep300	Mouse Erythro Leukaemic	GSE36030	GSM912921
Smc3	Mouse Erythro Leukaemic	GSE36030	GSM912923
Mxi1	Mouse Erythro Leukaemic	GSE36030	GSM912928
Chd2	Mouse Erythro Leukaemic	GSE36030	GSM912929
Rdbp	Mouse Erythro Leukaemic	GSE36030	GSM912932
Rad21	Mouse Erythro Leukaemic	GSE36030	GSM912933
Myc	Mouse Erythro Leukaemic	GSE36030	GSM912934
Rad21	Mouse Erythro Leukaemic	GSE36030	GSM912935
Gata1	Erythroid	GSE36589	GSM897224
Cbx7	Haematopoietic Progenitors	GSE36658	GSM898200
Cbx8	Haematopoietic Progenitors	GSE36658	GSM898201
Stat5a	T-Cells	GSE36882	GSM904760
Stat5b	T-Cells	GSE36882	GSM904761
Stat5a	T-Cells	GSE36882	GSM904766
Stat5b	T-Cells	GSE36882	GSM904767
Pax5	B-Cells	GSE38046	GSM932921-GSM932924
Pax5	B-Cells	GSE38046	GSM932925-GSM932931
Ikzf1	B-Cells	GSE38200	GSM1040573-GSM1040574
Ikzf1	B-Cells	GSE38200	GSM936199-GSM936200
Ikzf1	B-Cells	GSE38200	GSM936201-GSM936202
Sfpi1	Macrophages	GSE38377	GSM1022260
Sfpi1	Macrophages	GSE38377	GSM1022261
Sfpi1	Macrophages	GSE38377	GSM1022262
Sfpi1	Macrophages	GSE38377	GSM1022263
Sfpi1	Macrophages	GSE38377	GSM1022280
Sfpi1	Macrophages	GSE38377	GSM1022281
Sfpi1	Macrophages	GSE38377	GSM1022282
Sfpi1	Macrophages	GSE38377	GSM1022283
Stat6	Macrophages	GSE38377	GSM1022301
Stat6	Macrophages	GSE38377	GSM1022302
Stat6	Macrophages	GSE38377	GSM1022303
Stat6	Macrophages	GSE38377	GSM1022304
Stat6	Macrophages	GSE38377	GSM1022305
Sfpi1	Macrophages	GSE38377	GSM1022310
Sfpi1	Macrophages	GSE38377	GSM1022311
Sfpi1	Macrophages	GSE38377	GSM1022312
Sfpi1	Macrophages	GSE38377	GSM1022313
Stat1	Macrophages	GSE38377	GSM1022315

Stat1	Macrophages	GSE38377	GSM1022316
Stat1	Macrophages	GSE38377	GSM1022317
Junb	Macrophages	GSE38377	GSM1022318
Junb	Macrophages	GSE38377	GSM1022319
Sfpi1	Macrophages	GSE38377	GSM940924-GSM940925
Sfpi1	Macrophages	GSE38377	GSM940926
Sfpi1	Macrophages	GSE38377	GSM940927
Sfpi1	Macrophages	GSE38377	GSM940928
Sfpi1	Macrophages	GSE38377	GSM940929
Sfpi1	Macrophages	GSE38377	GSM940930
Sfpi1	Macrophages	GSE38377	GSM940931
Sfpi1	Macrophages	GSE38377	GSM940932
Sfpi1	Macrophages	GSE38377	GSM940933
Sfpi1	Macrophages	GSE38377	GSM940934
Sfpi1	Myeloid Progenitors	GSE38824	GSM1031977
Irf8	Myeloid Progenitors	GSE38824	GSM950325
Irf4	B-Cells	GSE39756	GSM978747
Irf4	B-Cells	GSE39756	GSM978748
Irf4	T-Cells	GSE39756	GSM978750
Irf4	T-Cells	GSE39756	GSM978751
Stat3	T-Cells	GSE39756	GSM978757
Batf	T-Cells	GSE39756	GSM978758
Batf	T-Cells	GSE39756	GSM978767
Irf4	T-Cells	GSE39756	GSM978768
Junb	T-Cells	GSE39756	GSM978769
Jun	T-Cells	GSE39756	GSM978770
Jund	T-Cells	GSE39756	GSM978771
Stat3	T-Cells	GSE39756	GSM978772
Runx1	ES-derived HSPC	GSE40235	GSM989013
Runx1	ES-derived HSPC	GSE40235	GSM989014
Fli1	ES-derived HSPC	GSE40235	GSM989015
Fli1	ES-derived HSPC	GSE40235	GSM989016
Cebpb	ES-derived HSPC	GSE40235	GSM989017
Tal1	ES-derived HSPC	GSE40235	GSM989018
Tal1	ES-derived HSPC	GSE40235	GSM989019
Fli1	ES-derived HSPC	GSE40235	GSM989023
Tal1	ES-derived HSPC	GSE40235	GSM989024
Ep300	T-Cells	GSE40463	GSM994508-GSM994529
Ep300	T-Cells	GSE40463	GSM994516-GSM994533
Ep300	T-Cells	GSE40463	GSM994520
Ep300	T-Cells	GSE40463	GSM994524
Stat1	T-Cells	GSE40463	GSM994528
Foxo1	T-Cells	GSE40656	GSM998924
Foxp3	T-Cells	GSE40684	GSM999179-GSM999180-GSM999182
Elf1	T-Cells	GSE40684	GSM999184

Elf1	T-Cells	GSE40684	GSM999185
Ets1	T-Cells	GSE40684	GSM999186
Ets1	T-Cells	GSE40684	GSM999187
Batf	T-Cells	GSE40918	GSM1004784-GSM1004785
Batf	T-Cells	GSE40918	GSM1004786-GSM1004787
Batf	T-Cells	GSE40918	GSM1004789-GSM1004791
Batf	T-Cells	GSE40918	GSM1004790-GSM1004792
Batf	T-Cells	GSE40918	GSM1004793
Batf	T-Cells	GSE40918	GSM1004794-GSM1004796
Batf	T-Cells	GSE40918	GSM1004795-GSM1004797
Maf	T-Cells	GSE40918	GSM1004799-GSM1004800
Ctcf	T-Cells	GSE40918	GSM1004802-GSM1004803
Ctcf	T-Cells	GSE40918	GSM1004804-GSM1004805
Etv6	T-Cells	GSE40918	GSM1004806-GSM1004807
Fosl2	T-Cells	GSE40918	GSM1004808
Fosl2	T-Cells	GSE40918	GSM1004809-GSM1004810
Hif1a	T-Cells	GSE40918	GSM1004819-GSM1004820
Irf4	T-Cells	GSE40918	GSM1004821
Irf4	T-Cells	GSE40918	GSM1004822
Irf4	T-Cells	GSE40918	GSM1004823-GSM1004824
Irf4	T-Cells	GSE40918	GSM1004825
Irf4	T-Cells	GSE40918	GSM1004826
Irf4	T-Cells	GSE40918	GSM1004827
Irf4	T-Cells	GSE40918	GSM1004828
Irf4	T-Cells	GSE40918	GSM1004829
Irf4	T-Cells	GSE40918	GSM1004830
Irf4	T-Cells	GSE40918	GSM1004831
Irf4	T-Cells	GSE40918	GSM1004833-GSM1004835
Irf4	T-Cells	GSE40918	GSM1004834-GSM1004836
Irf4	T-Cells	GSE40918	GSM1004837
Irf4	T-Cells	GSE40918	GSM1004838
Kdm6b	T-Cells	GSE40918	GSM1004839
Kdm6b	T-Cells	GSE40918	GSM1004840
Ep300	T-Cells	GSE40918	GSM1004841-GSM1004842
Ep300	T-Cells	GSE40918	GSM1004843
Ep300	T-Cells	GSE40918	GSM1004844
Ep300	T-Cells	GSE40918	GSM1004845
Ep300	T-Cells	GSE40918	GSM1004846
Ep300	T-Cells	GSE40918	GSM1004847
Ep300	T-Cells	GSE40918	GSM1004848
Ep300	T-Cells	GSE40918	GSM1004849-GSM1004850
Ep300	T-Cells	GSE40918	GSM1004851
Ep300	T-Cells	GSE40918	GSM1004852
Rorc	T-Cells	GSE40918	GSM1004855-GSM1004856
Stat3	T-Cells	GSE40918	GSM1004858
Stat3	T-Cells	GSE40918	GSM1004859

Stat3	T-Cells	GSE40918	GSM1004860-GSM1004861
Stat3	T-Cells	GSE40918	GSM1004863-GSM1004865
Stat3	T-Cells	GSE40918	GSM1004864-GSM1004866
Ctcf	Thymocytes	GSE41743	GSM1023416
Ctcf	Thymocytes	GSE41743	GSM1023418
Ctcf	B-Cells	GSE41743	GSM1023420
Ldb1	ES-derived HSPC	GSE43044	GSM1055553
Med1	B-Cells	GSE44288	GSM1038263
Ctcf	Haematopoietic Progenitors	GSE48086	GSM1167572
Tcf3	Haematopoietic Progenitors	GSE48086	GSM1167573
Ctcf	Mast	GSE48086	GSM1167574
Tcf3	Mast	GSE48086	GSM1167575
Erg	Mast	GSE48086	GSM1167576
Fli1	Mast	GSE48086	GSM1167577
Gata2	Mast	GSE48086	GSM1167578
Lmo2	Mast	GSE48086	GSM1167579
Meis1	Mast	GSE48086	GSM1167580
Sfpi1	Mast	GSE48086	GSM1167581
Runx1	Mast	GSE48086	GSM1167582
Tal1	Mast	GSE48086	GSM1167583
Mitf	Mast	GSE48086	GSM1167584
Fos	Mast	GSE48086	GSM1167585
Erg	Haematopoietic Progenitors	PMID20887958	PMID20887958
Gfi1b	Mouse Erythro Leukaemic	PMID22158964	PMID22158964

(B) Details of the 13 adipocyte samples used in Figure 4B.

**transcription factor**	**GSE**	**GSM**
Cebpb	GSE27826	GSM686970
Cebpb	GSE27826	GSM686971
Cebpb	GSE27826	GSM686972
Cebpb	GSE27826	GSM686973
Cebpd	GSE27826	GSM686974
Cebpd	GSE27826	GSM686975
Gcgr	GSE27826	GSM686976
Pparg	GSE27826	GSM686979
Pparg	GSE27826	GSM686980
Rxra	GSE27826	GSM686978
Stat5a	GSE27826	GSM686977
Rela	GSE35724	GSM873983
Ctcf	GSE20752	GSM535747

Supplementary Table 2. Table of unique motif-pairs with significant preferential spacing. Table used to generate 'circos' plot showing the number of unique motif-pairs (multiple offset value not counted).

motif_cluster	CCAAT	Cebp	CRE	CTCF	D-box	E-box	Ets	Forkhead	Gata	GC-box	Gfi1	HMG	Homeobox	Meis	Myb	Nuclear-receptor	Runt	TRE	Zinc-coordinating
**CCAAT**	3	0	8	0	0	5	11	0	0	0	3	0	0	0	0	0	0	0	17
**Cebp**	0	0	2	15	6	1	21	0	6	1	2	0	71	0	2	0	1	3	3
**CRE**	8	2	7	0	0	5	88	0	0	0	0	0	0	0	0	0	0	0	25
**CTCF**	0	15	0	0	0	7	54	0	0	0	0	0	67	0	0	0	12	6	24
**D-box**	0	6	0	0	3	0	17	0	0	0	0	0	2	0	1	0	0	2	0
**E-box**	5	1	5	7	0	47	67	0	28	2	1	1	3	0	1	0	2	5	41
**Ets**	11	21	88	54	17	67	126	17	27	24	7	8	182	14	13	10	22	41	86
**Forkhead**	0	0	0	0	0	0	17	2	0	0	0	2	0	0	0	0	0	0	0
**Gata**	0	6	0	0	0	28	27	0	10	8	0	0	24	2	8	0	12	17	10
**GC-box**	0	1	0	0	0	2	24	0	8	6	0	0	0	0	0	0	3	2	49
**Gfi1**	3	2	0	0	0	1	7	0	0	0	0	0	1	0	0	0	2	0	6
**HMG**	0	0	0	0	0	1	8	2	0	0	0	0	2	0	0	0	0	0	0
**Homeobox**	0	71	0	67	2	3	182	0	24	0	1	2	294	8	0	1	13	6	26
**Meis**	0	0	0	0	0	0	14	0	2	0	0	0	8	2	0	0	0	0	0
**Myb**	0	2	0	0	1	1	13	0	8	0	0	0	0	0	4	0	1	6	1
**Nuclear-receptor**	0	0	0	0	0	0	10	0	0	0	0	0	1	0	0	2	0	2	4
**Runt**	0	1	0	12	0	2	22	0	12	3	2	0	13	0	1	0	3	5	12
**TRE**	0	3	0	6	2	5	41	0	17	2	0	0	6	0	6	2	5	15	8
**Zinc-coordinating**	17	3	25	24	0	41	86	0	10	49	6	0	26	0	1	4	12	8	51

Supplementary Table 3. Probability matrices of 34 motifs that did not belong to any of the 19 motif clusters. PWMs have been trimmed as described in the methods section.

>CN0004.1

0.048482	0.034097	0.885456	0.031966
0.089505	0.612680	0.236548	0.061268
0.452850	0.408631	0.048482	0.090037
0.041023	0.192328	0.081513	0.685136
0.035695	0.205647	0.419286	0.339371
0.087906	0.773042	0.062334	0.076718
0.019180	0.156100	0.009590	0.815131
0.020245	0.002131	0.970698	0.006926
0.006926	0.004262	0.983484	0.005328
0.031433	0.008524	0.948322	0.011721
0.884923	0.023442	0.059670	0.031966
0.437933	0.122003	0.362813	0.077251
0.328716	0.115077	0.093767	0.462440
0.019712	0.101758	0.033564	0.844965
0.027704	0.011721	0.957912	0.002664
0.037826	0.049014	0.057539	0.855621
0.917954	0.009057	0.060202	0.012786
0.011188	0.019712	0.953649	0.015450
0.007991	0.015450	0.024507	0.952051
0.022376	0.466169	0.043154	0.468300
0.033564	0.575386	0.036228	0.354822

>CN0009.1

0.021483	0.146223	0.081081	0.751213
0.019404	0.932779	0.014553	0.033264
0.959806	0.009702	0.006930	0.023562
0.021483	0.018018	0.948025	0.012474
0.059598	0.701317	0.168399	0.070686
0.937630	0.017325	0.019404	0.025641
0.016632	0.934165	0.029799	0.019404
0.018018	0.913375	0.015246	0.053361
0.538462	0.139986	0.128898	0.192654
0.169092	0.265419	0.125433	0.440055
0.040887	0.014553	0.923077	0.021483

0.021483	0.016632	0.953569	0.008316
0.794872	0.129591	0.040194	0.035343
0.011781	0.798337	0.144144	0.045738
0.970894	0.004158	0.011781	0.013167
0.042273	0.018018	0.928621	0.011088
0.137214	0.600832	0.130977	0.130977
0.214137	0.040194	0.424809	0.320859
0.139293	0.573805	0.209286	0.077616
0.102564	0.722107	0.031185	0.144144

>MA0014.1

0.333333	0.000000	0.666667	0.000000
0.333333	0.250000	0.250000	0.166667
0.083333	0.166667	0.416667	0.333333
0.166667	0.583333	0.083333	0.166667
0.583333	0.166667	0.083333	0.166667
0.166667	0.416667	0.250000	0.166667
0.000000	0.250000	0.166667	0.583333
0.083333	0.166667	0.666667	0.083333
0.500000	0.083333	0.250000	0.166667
0.500000	0.000000	0.166667	0.333333
0.000000	0.000000	1.000000	0.000000
0.166667	0.666667	0.083333	0.083333
0.250000	0.000000	0.750000	0.000000
0.083333	0.000000	0.333333	0.583333
0.500000	0.083333	0.416667	0.000000
0.416667	0.083333	0.416667	0.083333
0.166667	0.833333	0.000000	0.000000
0.166667	0.416667	0.416667	0.000000
0.416667	0.000000	0.500000	0.083333

>MA0019.1

0.000000	0.025641	0.000000	0.974359
0.000000	0.000000	0.974359	0.025641
0.102564	0.846154	0.000000	0.051282
0.974359	0.025641	0.000000	0.000000
0.923077	0.051282	0.025641	0.000000

0.000000	0.153846	0.000000	0.846154
0.358974	0.435897	0.128205	0.076923
0.102564	0.589744	0.230769	0.076923
0.000000	0.666667	0.153846	0.179487

>MA0050.1

0.300000	0.200000	0.500000	0.000000
0.950000	0.000000	0.050000	0.000000
0.950000	0.000000	0.000000	0.050000
1.000000	0.000000	0.000000	0.000000
0.250000	0.150000	0.550000	0.050000
0.000000	0.500000	0.000000	0.500000
0.050000	0.050000	0.900000	0.000000
1.000000	0.000000	0.000000	0.000000
0.950000	0.050000	0.000000	0.000000
1.000000	0.000000	0.000000	0.000000
0.050000	0.650000	0.300000	0.000000
0.050000	0.650000	0.050000	0.250000

>MA0067.1

0.225806	0.032258	0.677419	0.064516
0.096774	0.064516	0.000000	0.838710
0.000000	0.903226	0.032258	0.064516
0.838710	0.032258	0.096774	0.032258
0.064516	0.548387	0.032258	0.354839
0.064516	0.000000	0.612903	0.322581

>MA0068.1

0.952381	0.000000	0.047619	0.000000
0.761905	0.095238	0.047619	0.095238
0.523810	0.047619	0.047619	0.380952
0.619048	0.047619	0.142857	0.190476
0.523810	0.142857	0.047619	0.285714
0.285714	0.047619	0.095238	0.571429
0.428571	0.047619	0.047619	0.476190
0.238095	0.142857	0.285714	0.333333
0.238095	0.523810	0.047619	0.190476

0.285714	0.523810	0.190476	0.000000
0.333333	0.333333	0.238095	0.095238
0.380952	0.333333	0.095238	0.190476
0.285714	0.238095	0.047619	0.428571
0.476190	0.238095	0.190476	0.095238
0.190476	0.380952	0.190476	0.238095
0.142857	0.285714	0.190476	0.380952
0.333333	0.380952	0.142857	0.142857
0.190476	0.428571	0.142857	0.238095
0.428571	0.285714	0.095238	0.190476
0.238095	0.333333	0.238095	0.190476
0.238095	0.285714	0.095238	0.380952
0.333333	0.523810	0.000000	0.142857

>MA0079.1

0.000000	0.000000	1.000000	0.000000
0.000000	0.000000	1.000000	0.000000
0.000000	0.625000	0.250000	0.125000
0.250000	0.000000	0.500000	0.250000
0.000000	0.125000	0.625000	0.250000
0.000000	0.000000	0.750000	0.250000
0.125000	0.125000	0.750000	0.000000
0.250000	0.000000	0.000000	0.750000

>MA0088.1

0.000000	0.300000	0.200000	0.500000
0.200000	0.100000	0.000000	0.700000
0.100000	0.700000	0.000000	0.200000
0.000000	1.000000	0.000000	0.000000
0.000000	1.000000	0.000000	0.000000
0.900000	0.100000	0.000000	0.000000
0.000000	0.100000	0.400000	0.500000
0.400000	0.400000	0.200000	0.000000
0.600000	0.200000	0.200000	0.000000
0.000000	0.000000	0.100000	0.900000
0.000000	0.100000	0.600000	0.300000
0.000000	1.000000	0.000000	0.000000

0.400000	0.500000	0.000000	0.100000
0.300000	0.100000	0.100000	0.500000
0.200000	0.300000	0.000000	0.500000
0.100000	0.100000	0.700000	0.100000
0.300000	0.600000	0.100000	0.000000

>MA0108.1

0.041131	0.118252	0.046272	0.794344
0.904884	0.000000	0.005141	0.089974
0.007712	0.025707	0.005141	0.961440
0.910026	0.000000	0.012853	0.077121
0.688946	0.000000	0.000000	0.311054
0.949868	0.007916	0.026385	0.015831
0.570694	0.005141	0.113111	0.311054

>MA0108.2

0.041131	0.118252	0.046272	0.794344
0.904884	0.000000	0.005141	0.089974
0.007712	0.025707	0.005141	0.961440
0.910026	0.000000	0.012853	0.077121
0.688946	0.000000	0.000000	0.311054
0.925450	0.007712	0.051414	0.015424
0.570694	0.005141	0.113111	0.311054

>MA0123.1

0.000000	1.000000	0.000000	0.000000
0.244898	0.000000	0.755102	0.000000
0.000000	0.408163	0.591837	0.000000
0.000000	0.469388	0.020408	0.510204
0.020408	0.061224	0.918367	0.000000
0.000000	0.918367	0.081633	0.000000
0.102041	0.571429	0.122449	0.204082
0.061224	0.510204	0.224490	0.204082
0.061224	0.632653	0.102041	0.204082

>MA0146.1

0.020790	0.617464	0.299376	0.062370

0.012474	0.752599	0.004158	0.230769
0.062370	0.259875	0.378378	0.299376
0.397089	0.320166	0.251559	0.031185
0.018711	0.004158	0.975052	0.002079
0.000000	0.006237	0.991684	0.002079
0.002079	0.997921	0.000000	0.000000
0.000000	0.997921	0.000000	0.002079
0.000000	0.004158	0.000000	0.995842

>MA0158.1

0.133333	0.866667	0.000000	0.000000
0.437500	0.000000	0.312500	0.250000
0.000000	0.437500	0.312500	0.250000
0.375000	0.000000	0.062500	0.562500
0.875000	0.000000	0.000000	0.125000
0.875000	0.000000	0.125000	0.000000
0.000000	0.000000	0.000000	1.000000
0.000000	0.062500	0.375000	0.562500

>MA0213.1

0.100000	0.500000	0.400000	0.000000
0.000000	0.400000	0.000000	0.600000
0.000000	0.000000	1.000000	0.000000
0.000000	0.000000	1.000000	0.000000
0.000000	1.000000	0.000000	0.000000
0.100000	0.000000	0.900000	0.000000
0.100000	0.800000	0.000000	0.100000
0.000000	0.500000	0.100000	0.400000

>MA0231.1

0.000000	0.000000	0.000000	1.000000
1.000000	0.000000	0.000000	0.000000
1.000000	0.000000	0.000000	0.000000
0.045455	0.454545	0.090909	0.409091
0.181818	0.227273	0.136364	0.454545
0.863636	0.000000	0.136364	0.000000

>MA0243.1

0.214286	0.000000	0.571429	0.214286
0.571429	0.071429	0.071429	0.285714
0.285714	0.571429	0.071429	0.071429
1.000000	0.000000	0.000000	0.000000
0.071429	0.071429	0.000000	0.857143
0.214286	0.000000	0.071429	0.714286
0.000000	0.571429	0.000000	0.428571

>MA0275.1

0.000000	0.760000	0.000000	0.240000
0.000000	1.000000	0.000000	0.000000
0.000000	0.000000	1.000000	0.000000
0.000000	0.000000	1.000000	0.000000
0.727273	0.111111	0.161616	0.000000
0.530000	0.000000	0.000000	0.470000

>MA0280.1

0.000000	1.000000	0.000000	0.000000
0.000000	1.000000	0.000000	0.000000
0.000000	0.000000	1.000000	0.000000
0.000000	0.000000	1.000000	0.000000
0.660000	0.130000	0.210000	0.000000

>MA0373.1

0.232323	0.000000	0.676768	0.090909
0.000000	0.000000	1.000000	0.000000
0.000000	0.000000	0.120000	0.880000
0.000000	0.000000	1.000000	0.000000
0.000000	0.000000	1.000000	0.000000
0.000000	1.000000	0.000000	0.000000
0.130000	0.000000	0.870000	0.000000

>MA0406.1

0.000000	0.950000	0.050000	0.000000
1.000000	0.000000	0.000000	0.000000
0.050000	0.000000	0.000000	0.950000

0.000000	0.000000	0.000000	1.000000
0.000000	0.910000	0.090000	0.000000
0.000000	0.680000	0.000000	0.320000

>MA0424.1

0.020000	0.170000	0.100000	0.710000
0.000000	0.770000	0.150000	0.080000
0.000000	1.000000	0.000000	0.000000
0.000000	0.000000	1.000000	0.000000
0.000000	0.000000	1.000000	0.000000
0.690000	0.310000	0.000000	0.000000

>MA0427.1

0.020000	0.940000	0.020000	0.020000
0.000000	1.000000	0.000000	0.000000
0.000000	0.070707	0.929293	0.000000
0.050000	0.010000	0.930000	0.010000

>PF0004.1

1.000000	0.000000	0.000000	0.000000
0.000000	1.000000	0.000000	0.000000
0.000000	0.000000	0.000000	1.000000
1.000000	0.000000	0.000000	0.000000
0.000000	0.990164	0.000000	0.009836
0.896721	0.000000	0.103279	0.000000
0.556148	0.094262	0.069262	0.280328
0.129508	0.312705	0.126639	0.431148
0.038525	0.057787	0.028689	0.875000
0.000000	1.000000	0.000000	0.000000
0.000000	1.000000	0.000000	0.000000
0.000000	1.000000	0.000000	0.000000
0.809016	0.000000	0.190984	0.000000

>PF0018.1

0.000000	0.000000	0.000000	1.000000
0.000000	1.000000	0.000000	0.000000
1.000000	0.000000	0.000000	0.000000

0.063139	0.529927	0.250730	0.156204
0.189781	0.241606	0.532117	0.036496
0.000000	0.000000	0.000000	1.000000
0.000000	0.000000	1.000000	0.000000
1.000000	0.000000	0.000000	0.000000
0.000000	0.724453	0.000000	0.275547

>PF0039.1

0.000000	0.000000	0.000000	1.000000
0.000000	1.000000	0.000000	0.000000
0.000000	1.000000	0.000000	0.000000
0.000000	1.000000	0.000000	0.000000
0.646484	0.000000	0.353516	0.000000
0.122070	0.133789	0.583984	0.160156
0.228516	0.502930	0.225586	0.042969
0.709961	0.000000	0.290039	0.000000
0.000000	0.000000	0.000000	1.000000
0.000000	0.000000	1.000000	0.000000
0.000000	1.000000	0.000000	0.000000

>PF0070.1

0.000000	1.000000	0.000000	0.000000
0.000000	0.000000	1.000000	0.000000
0.000000	0.000000	0.000000	1.000000
0.000000	0.457143	0.542857	0.000000
1.000000	0.000000	0.000000	0.000000
0.000000	1.000000	0.000000	0.000000
0.000000	0.000000	1.000000	0.000000

>PF0074.1

0.000000	0.000000	1.000000	0.000000
0.000000	0.000000	1.000000	0.000000
1.000000	0.000000	0.000000	0.000000
0.171533	0.828467	0.000000	0.000000
0.000000	0.000000	0.000000	1.000000
0.496350	0.312044	0.085766	0.105839
0.031022	0.797445	0.074818	0.096715

0.600365	0.067518	0.246350	0.085766
0.330292	0.094891	0.156934	0.417883
0.069343	0.301095	0.120438	0.509124
0.000000	0.000000	0.000000	1.000000
0.000000	1.000000	0.000000	0.000000
0.000000	1.000000	0.000000	0.000000
0.000000	0.945255	0.000000	0.054745

>PF0085.1

0.000000	0.625000	0.375000	0.000000
0.000000	0.000000	0.000000	1.000000
0.000000	0.000000	0.000000	1.000000
0.000000	0.000000	0.000000	1.000000
0.000000	1.000000	0.000000	0.000000
0.632911	0.000000	0.367089	0.000000
0.099684	0.360759	0.283228	0.256329
0.000000	0.000000	0.000000	1.000000
0.000000	0.000000	0.000000	1.000000
0.000000	0.000000	0.000000	1.000000

>PF0092.1

0.000000	0.000000	0.000000	1.000000
0.000000	0.000000	1.000000	0.000000
0.000000	1.000000	0.000000	0.000000
0.000000	0.000000	0.000000	1.000000
0.000000	0.000000	1.000000	0.000000
1.000000	0.000000	0.000000	0.000000
0.000000	0.592637	0.000000	0.407363

>PF0108.1

1.000000	0.000000	0.000000	0.000000
1.000000	0.000000	0.000000	0.000000
0.000000	1.000000	0.000000	0.000000
0.211921	0.000000	0.000000	0.788079
0.844371	0.000000	0.000000	0.155629
0.000000	1.000000	0.000000	0.000000
1.000000	0.000000	0.000000	0.000000

1.000000	0.000000	0.000000	0.000000
0.259934	0.375828	0.168874	0.195364
0.000000	0.000000	0.271523	0.728477

>PF0128.1

0.322034	0.000000	0.677966	0.000000
0.000000	0.572034	0.000000	0.427966
0.000000	1.000000	0.000000	0.000000
1.000000	0.000000	0.000000	0.000000
0.000000	1.000000	0.000000	0.000000
0.330508	0.262712	0.245763	0.161017
0.330508	0.161017	0.355932	0.152542
0.330508	0.000000	0.669492	0.000000
0.389831	0.165254	0.381356	0.063559
0.194915	0.228814	0.317797	0.258475
0.444915	0.000000	0.555085	0.000000
0.203390	0.216102	0.453390	0.127119
0.000000	1.000000	0.000000	0.000000
1.000000	0.000000	0.000000	0.000000
0.000000	0.000000	1.000000	0.000000

>PF0156.1

0.000000	0.866438	0.000000	0.133562
0.400685	0.000000	0.599315	0.000000
0.000000	1.000000	0.000000	0.000000
0.000000	1.000000	0.000000	0.000000
1.000000	0.000000	0.000000	0.000000
0.000000	0.000000	0.832192	0.167808
0.133562	0.232877	0.578767	0.054795
0.013699	0.160959	0.472603	0.352740
0.000000	0.000000	1.000000	0.000000
0.095890	0.325342	0.520548	0.058219
0.000000	1.000000	0.000000	0.000000
0.000000	0.000000	1.000000	0.000000
0.000000	1.000000	0.000000	0.000000

>POL012.1

0.041131	0.118252	0.046272	0.794344
0.904884	0.000000	0.005141	0.089974
0.007712	0.025707	0.005141	0.961440
0.910026	0.000000	0.012853	0.077121
0.688946	0.000000	0.000000	0.311054
0.949868	0.007916	0.026385	0.015831
0.570694	0.005141	0.113111	0.311054

Supplementary Table 4. GREAT analysis performed on candidate motif-pair regions (see Supplementary Methods).

(A) E-box + GATA

# Term Name	Binom FDR Q-Val	Binom Fold Enrichment	Binom Region Set Coverage	Hyper Observed Gene Hits	Hyper Gene Set Coverage
**(I) GO Biological Process**					
cellular response to dexamethasone stimulus	0.018549	10.16833	0.01227	5	0.005945
hemopoiesis	0.018466	2.033245	0.07771	33	0.039239
positive regulation of epithelial to mesenchymal transition	0.024075	5.334056	0.018405	7	0.008323
myeloid cell differentiation	0.022971	2.927874	0.03681	15	0.017836
actin cytoskeleton organization	0.024511	2.392634	0.051125	23	0.027348
actin filament-based process	0.030388	2.309659	0.051125	23	0.027348
response to dexamethasone stimulus	0.039522	6.087291	0.014315	6	0.007134
positive regulation of cell morphogenesis involved in differentiation	0.043458	4.497834	0.018405	7	0.008323
**(II) Mouse Phenotype**					
abnormal megakaryocyte progenitor cell morphology	0.006682	2.858191	0.04908	21	0.02497
anemia	0.011017	2.321451	0.06544	28	0.033294
enlarged spleen	0.016468	2.06827	0.07771	34	0.040428
extramedullary hematopoiesis	0.032928	2.797764	0.03681	16	0.019025
decreased guard hair length	0.032998	15.62336	0.00818	3	0.003567
lethargy	0.040308	3.00951	0.030675	14	0.016647
abnormal guard hair length	0.041848	14.12181	0.00818	3	0.003567
**(III) Disease Ontology**					
leukopenia	0.006465	5.130638	0.022495	9	0.010702
osteoporosis	0.01145	2.933664	0.038855	16	0.019025
pancytopenia	0.017648	16.73165	0.00818	3	0.003567
blood coagulation disease	0.01698	2.003634	0.06953	30	0.035672
hemorrhagic disease	0.017444	2.013445	0.067485	29	0.034483
bone remodeling disease	0.022729	2.446521	0.042945	18	0.021403
plasmacytoma	0.025461	2.199607	0.051125	21	0.02497
labyrinthine disease	0.025053	6.891771	0.01227	5	0.005945
mature B-cell lymphocytic neoplasm	0.024069	2.168226	0.051125	21	0.02497
pilomatrixoma	0.025479	8.379612	0.010225	3	0.003567
otosclerosis	0.025233	8.342288	0.010225	4	0.004756
multiple myeloma	0.026165	2.173058	0.04908	20	0.023781
herpes simplex	0.03889	3.190207	0.022495	11	0.01308

(B) Ets + Homeobox

# Term Name	Binom FDR Q-Val	Binom Fold Enrichment	Binom Region Set Coverage	Hyper Observed Gene Hits	Hyper Gene Set Coverage
**(I) GO Biological Process**					
regulation of protein metabolic process	0.001868	2.015138	0.194346	55	0.109127
positive regulation of protein metabolic process	0.006869	2.459775	0.102474	31	0.061508
positive regulation of cellular protein metabolic process	0.046168	2.297793	0.084806	26	0.051587
**(II) Mouse Phenotype**					
abnormal leukocyte morphology	4.36E-05	2.024923	0.233216	70	0.138889
abnormal immune system cell morphology	4.16E-05	2.012354	0.233216	70	0.138889
abnormal bone marrow cell morphology/development	9.73E-05	2.136191	0.194346	60	0.119048
abnormal mononuclear cell morphology	0.000101	2.033366	0.212014	63	0.125
decreased white fat cell lipid droplet size	0.001025	28.68817	0.017668	4	0.007937
abnormal brown fat cell morphology	0.001096	10.32212	0.028269	7	0.013889
abnormal lymphocyte morphology	0.001039	2.011566	0.176678	53	0.105159
abnormal white fat cell lipid droplet size	0.001003	26.07358	0.017668	4	0.007937
impaired wound healing	0.001467	7.867854	0.031802	7	0.013889
abnormal fat cell morphology	0.001647	4.265499	0.053004	14	0.027778
abnormal brown adipose tissue morphology	0.002027	4.430251	0.04947	13	0.025794
abnormal myeloid leukocyte morphology	0.002258	2.271005	0.123675	37	0.073413
abnormal myeloblast morphology/development	0.002397	2.03313	0.151944	47	0.093254
increased hematopoietic cell number	0.002846	2.07633	0.141343	42	0.083333
abnormal brown fat cell lipid droplet size	0.004114	11.46762	0.021201	5	0.009921
abnormal white fat cell morphology	0.004279	7.238586	0.028269	7	0.013889
abnormal oxygen consumption	0.005659	4.761935	0.038869	10	0.019841
increased leukocyte cell number	0.005635	2.085572	0.127209	37	0.073413
abnormal epidermal layer morphology	0.006014	2.77352	0.074205	21	0.041667
abnormal inguinal fat pad morphology	0.006769	6.59653	0.028269	8	0.015873
abnormal osteoclast differentiation	0.009022	4.40422	0.038869	11	0.021825
abnormal granulocyte morphology	0.00961	2.57882	0.077739	24	0.047619
decreased susceptibility to diet-induced obesity	0.009447	4.77983	0.035336	9	0.017857
increased oxygen consumption	0.010642	5.252647	0.031802	8	0.015873
abnormal hematopoietic system physiology	0.010629	2.620697	0.074205	21	0.041667
increased energy expenditure	0.011548	4.220115	0.038869	10	0.019841
abnormal antigen presenting cell morphology	0.013453	2.065243	0.113074	32	0.063492
abnormal circulating tumor necrosis factor level	0.013396	5.735106	0.028269	8	0.015873
abnormal phagocyte morphology	0.020678	2.121676	0.09894	30	0.059524

abnormal respiratory quotient	0.022478	6.039151	0.024735	6	0.011905
decreased susceptibility to weight gain	0.023309	4.111337	0.035336	9	0.017857
arrested B cell differentiation	0.023112	4.522462	0.031802	9	0.017857
abnormal B cell morphology	0.025438	2.046325	0.102474	30	0.059524
abnormal immature B cell morphology	0.031674	2.866402	0.053004	15	0.029762
abnormal B cell differentiation	0.032174	2.191086	0.084806	24	0.047619
abnormal energy expenditure	0.03909	3.071381	0.045936	12	0.02381
abnormal tail bud morphology	0.039629	8.179951	0.017668	5	0.009921
abnormal circulating cytokine level	0.045474	3.013131	0.045936	14	0.027778
abnormal innate immunity	0.04631	2.123286	0.084806	25	0.049603
enlarged spleen	0.046567	2.163087	0.081272	24	0.047619
increased lung elastance	0.047291	61.00541	0.007067	2	0.003968
abnormal adipose tissue physiology	0.048841	5.068821	0.024735	7	0.013889
**(III) Disease Ontology**					
papillary epithelial neoplasm	0.04846	3.435776	0.053004	15	0.029762

(C) Ets + E-box

# Term Name	Binom FDR Q-Val	Binom Fold Enrichment	Binom Region Set Coverage	Hyper Observed Gene Hits	Hyper Gene Set Coverage
**(I) GO Biological Process**					
wound healing	0.028224	2.282774	0.044728	24	0.023121
regulation of T cell mediated immunity	0.028396	4.733884	0.015974	9	0.008671
lymphocyte activation	0.03473	2.052715	0.054313	33	0.031792
regulation of generation of precursor metabolites and energy	0.033581	4.187778	0.017572	9	0.008671
**(II) Mouse Phenotype**					
abnormal lymphocyte morphology	1.34e-10	2.109766	0.185304	115	0.11079
abnormal lymphopoiesis	1.98e-10	2.248061	0.158147	97	0.093449
abnormal mononuclear cell differentiation	1.82e-10	2.235983	0.158147	97	0.093449
abnormal leukopoiesis	5.68e-10	2.15046	0.162939	100	0.096339
abnormal myeloblast morphology/development	4.82e-10	2.158888	0.161342	99	0.095376
abnormal bone marrow cell morphology/development	5.38e-10	2.019237	0.183706	114	0.109827
abnormal T cell morphology	1.52e-9	2.203965	0.146965	89	0.085742
abnormal T cell differentiation	2.24e-8	2.292624	0.121406	73	0.070328
abnormal lymphocyte cell number	3.45e-8	2.030927	0.151757	94	0.090559
decreased lymphocyte cell number	1.14e-7	2.088979	0.13099	80	0.077071
thymus hypoplasia	1.81e-7	3.356011	0.057508	34	0.032755
abnormal T cell number	4.67e-7	2.126529	0.116613	69	0.066474
abnormal thymus morphology	5.43e-7	2.238933	0.103834	59	0.05684
small thymus	5.31e-7	2.67669	0.07508	44	0.042389
abnormal T cell physiology	5.64e-7	2.183996	0.108626	63	0.060694
abnormal thymus size	7.19e-7	2.491305	0.083067	47	0.045279

increased T cell number	1.53E-06	2.539101	0.076677	45	0.043353
abnormal B cell morphology	3.23E-06	2.105391	0.105431	68	0.065511
increased lymphocyte cell number	3.28E-06	2.21763	0.094249	57	0.054913
abnormal response to injury	7.15E-06	2.259712	0.086262	49	0.047206
**(III) Disease Ontology**					
liver cirrhosis	0.001501	2.445461	0.044728	26	0.025048
oligodendroglioma	0.00319	7.464912	0.011182	6	0.00578
myocardial infarction	0.007685	2.05742	0.049521	29	0.027938
disseminated intravascular coagulation	0.010547	5.7212	0.011182	6	0.00578
vitreous disease	0.025729	10.01913	0.00639	4	0.003854
hyperglycemia	0.031306	2.385718	0.027157	15	0.014451
collagen disease	0.040636	2.004473	0.036741	22	0.021195
paranoid schizophrenia	0.042902	8.049612	0.00639	4	0.003854
colon carcinoma	0.043726	2.105933	0.031949	20	0.019268
IgA glomerulonephritis	0.0438	2.746725	0.019169	12	0.011561

Supplementary Table 5. Probability matrices and logos of candidate motif pairs generated from aligned sequences of the 3 candidate motif-pair regions.

(A) E-box + GATA

A	0.536	0.334	0.039	0.973	0.027	0.149	0.000	0.000
C	0.192	0.229	0.961	0.016	0.133	0.611	0.000	0.004
G	0.234	0.376	0.000	0.006	0.630	0.227	0.012	0.982
T	0.038	0.061	0.000	0.004	0.211	0.012	0.988	0.014
A	0.098	0.249	0.243	0.241	0.276	0.270	0.301	0.196
C	0.323	0.196	0.196	0.280	0.227	0.239	0.157	0.364
G	0.278	0.223	0.299	0.260	0.227	0.278	0.305	0.278
T	0.301	0.331	0.262	0.219	0.270	0.213	0.237	0.162
A	0.624	0.002	0.998	0.004	0.998	0.797	0.003	0.000
C	0.029	0.000	0.000	0.000	0.000	0.000	0.311	0.000
G	0.057	0.996	0.000	0.002	0.002	0.119	0.685	0.938
T	0.290	0.002	0.002	0.994	0.000	0.084	0.000	0.063

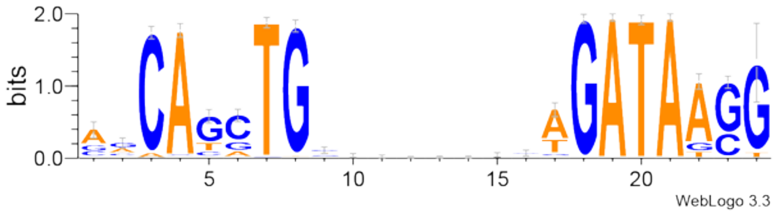

(B) Ets + Homeobox

A	0.033	0.028	0.961	1.000	0.018	0.032	0.637	0.007
C	0.381	0.021	0.021	0.000	0.000	0.014	0.011	0.715
G	0.095	0.004	0.014	0.000	0.028	0.408	0.327	0.106
T	0.490	0.947	0.004	0.000	0.954	0.546	0.025	0.173
A	0.028	0.000	0.000	0.018	0.036	0.012	0.333	
C	0.000	0.000	0.951	0.944	0.111	0.333	0.000	
G	0.004	0.000	0.000	0.000	0.089	0.532	0.000	
T	0.968	1.000	0.049	0.039	0.764	0.123	0.667	

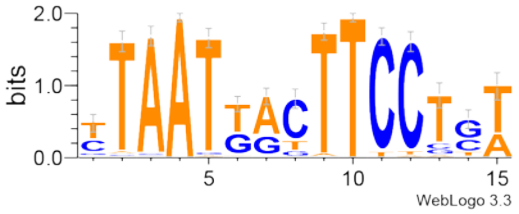

(C) Ets + E-box

A	0.655	0.052	0.743	0.000	0.049	1.000	0.944	0.287
C	0.122	0.695	0.151	0.000	0.000	0.000	0.002	0.016
G	0.000	0.229	0.084	0.986	0.951	0.000	0.037	0.643
T	0.223	0.024	0.022	0.014	0.000	0.000	0.018	0.054

A	0.000	0.997	0.002	0.008	0.002	0.000	0.047	0.009
C	0.998	0.002	0.312	0.795	0.002	0.000	0.377	0.358
G	0.000	0.000	0.678	0.196	0.000	0.998	0.302	0.009
T	0.002	0.002	0.008	0.002	0.997	0.002	0.274	0.623

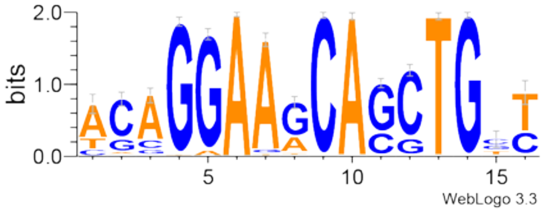

Supplement: SUPPLEMENTARY DATA [file supp_gku1254_nar-02141-z-2014-File008.pdf]
